# A statistically inferred microRNA network identifies breast cancer target miR-940 as an actin cytoskeleton regulator

**DOI:** 10.1038/srep08336

**Published:** 2015-02-12

**Authors:** Ricky Bhajun, Laurent Guyon, Amandine Pitaval, Eric Sulpice, Stéphanie Combe, Patricia Obeid, Vincent Haguet, Itebeddine Ghorbel, Christian Lajaunie, Xavier Gidrol

**Affiliations:** 1 Univ. Grenoble Alpes, iRTSV-BGE, F-38000 Grenoble, France; 2 CEA, iRTSV-BGE, F-38000 Grenoble, France; 3 INSERM, BGE, F-38000 Grenoble, France; 4 Center for Computational Biology - CBIO, Mines ParisTech, F-77300 Fontainebleau, France; 5 Institut Curie, F-75248 Paris, France; 6 INSERM, U900, F-75248 Paris, France

## Abstract

MiRNAs are key regulators of gene expression. By binding to many genes, they create a complex network of gene co-regulation. Here, using a network-based approach, we identified miRNA hub groups by their close connections and common targets. In one cluster containing three miRNAs, miR-612, miR-661 and miR-940, the annotated functions of the co-regulated genes suggested a role in small GTPase signalling. Although the three members of this cluster targeted the same subset of predicted genes, we showed that their overexpression impacted cell fates differently. miR-661 demonstrated enhanced phosphorylation of myosin II and an increase in cell invasion, indicating a possible oncogenic miRNA. On the contrary, miR-612 and miR-940 inhibit phosphorylation of myosin II and cell invasion. Finally, expression profiling in human breast tissues showed that miR-940 was consistently downregulated in breast cancer tissues

MicroRNAs are a class of endogenous, small (19–25 nucleotides), single-stranded non-coding RNAs that regulate gene expression in all eukaryotic organisms. In metazoans, microRNAs most commonly bind to the 3′ untranslated region (3′UTR) of their mRNA target transcript and cause translational repression and/or mRNA degradation. Every microRNA is predicted to regulate from a dozen to thousands of genes, including transcription factors. This fine-tuning of protein expression is known to be involved in many physiological processes, such as development, apoptosis, signal transduction and even cancer progression^1^,^2^. More than 2,000 mature human microRNAs are listed in the 20^th^ release of miRBase: http://www.mirbase.org (2014) (Date of access:19/08/2013), and some authors hypothesise that the majority of human genes are regulated by microRNAs^3^.

Since their discovery in 1993^4^, a fair understanding of their role in animal development and in the onset and progression of diseases^2^, as well as of their potential use in therapies^5^, has been gathered. However, the cooperative behaviour of microRNAs is still under investigation. A growing body of experimental evidence suggests that microRNAs can regulate genes through complementarity, meaning that microRNAs can act together to regulate individual genes or groups of genes involved in similar processes^6^. For example, Hu and co-workers demonstrated that transducing a cocktail of precursor microRNAs (miR-21, miR-24 and miR-221) can result in more effective engraftment of transplanted cardiac progenitor cells^7^. Consistent with these discoveries, Zhu *et al.* demonstrated that miR-21 and miR-221 coregulate 56 gene ontology (GO) processes^8^. In the same study, the authors also showed that cotransfection of miR-1 and miR-21 increases H_2_O_2_-induced myocardial apoptosis and oxidative stress.

These recent findings support the idea of microRNA-mediated cooperative regulation but also argue for the use of systemic approaches, notably based on graph theory, to decipher individual and complementary roles of microRNAs. Some work has been conducted to use recent high-throughput experiment-derived data sets to infer microRNA synergistic relationships^9^,^10^,^11^,^12^. Herein, we present a microRNA network based on target similarities among microRNAs to infer clusters of microRNAs. Clusters are defined as groups of microRNAs sharing a set of common targets, predicted by either DIANA-microT v3^13^ or TargetScan v6.2^14^. Some authors have used GO enrichment analysis as a confirmatory tool for their clustering approach^11^. In our case, GO enrichment is not used to infer networks but as a way to estimate the probable metabolic pathway(s) a cluster of microRNAs could co-regulate. Moreover, the novelty of our approach is to consider not only clusters of microRNAs but also “microRNA hubs”, *i.e.,* highly connected microRNAs presenting a crucial role in the network. We further defined these interconnected microRNA hubs as “assorted clubs” of microRNAs.

This target-based microRNA network shows many similarities with known biological networks and is constructed around two microRNA assorted clubs. These two clubs influence the overall shape of the network and thus the microRNAs connected to them. One of the assorted clubs was predicted to play a role in small GTPase signalling. Small GTPase proteins are divided in two subfamilies: members of the Ras subfamily which regulate cell proliferation and differentiation, and members of the Rho subfamily which control cytoskeleton and cell motility but can also act on proliferation^15^. Strikingly, all three microRNAs in the club, miR-612, miR-661, and miR-940, efficiently downregulate small GTPase signalling. However, their cellular function diverges showing that microRNAs acting on similar pathways can lead to opposite outputs. Indeed, Transwell assays and wound healing assays demonstrate that overexpression of miR-661 leads to a dramatic increase in cell motility, while miR-612 and miR-940 reduce this capacity. In addition, miR-940 was found consistently downregulated in breast cancer tissues, indicating a putative role of this microRNA in cancer progression.

## Results

### A target-based microRNA complementary network

To evaluate how microRNAs could act together on cellular processes, we intended to infer networks based on microRNA target sharing. We used the genome-wide DIANA-microT v3.0 prediction database^13^, comprising 555 human microRNAs, 18,986 genes and nearly 2 million interactions. We considered only *Homo sapiens* interactions and did not take into account any score but rather used all available information. In consequence, around 60% microRNAs are predicted to have between 2,000 and 5,000 different target sites and four microRNAs (miR-495, miR-548c-3p, miR-590-3p and miR-603) are predicted to exhibit affinity towards more than 10,000 target sites – which represents around 6000 genes (Supplementary Figures S1a & S1b). Furthermore, 193 genes are targeted by more than 293 microRNAs (Supplementary Figure S1c), which is the 99^th^ quantile of the microRNA-mediated target regulation histogram. These 193 proteins will therefore be referred to as gene (or protein) hubs^9^.

**Figure 1:**
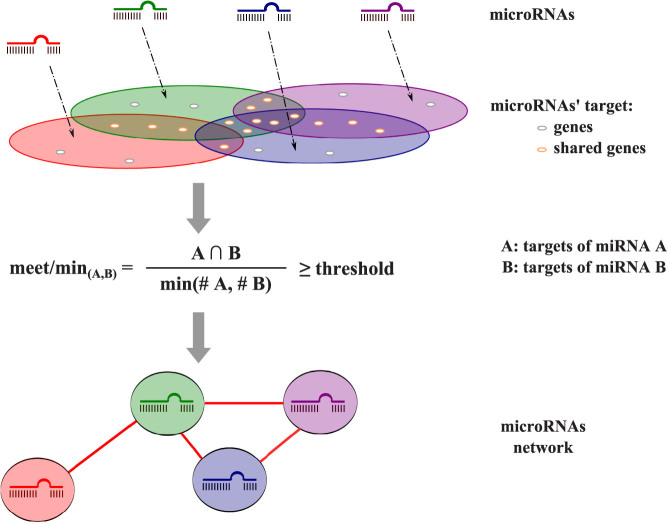
Construction of the microRNA network. The meet/min metric measures target coverage between microRNAs. A threshold (0.5 is chosen throughout the article) is imposed on the meet/min edges, thus defining a binary network of microRNAs that share common targets. #: number of.

Following Shalgi *et al.*^9^, we built a target-based microRNA networks based on the idea that if two microRNAs share a common set of genes, they could act on the same pathway(s) and compensate for each other, or act complementarily, on this pathway. Considering the DIANA-microT v3.0 predictions, each node of the network corresponds to a microRNA and each edge between two nodes to the proportion of shared targets between two microRNAs (Figure 1). We used the meet/min metric (or Simpson index) to infer the strength of the edge between two microRNAs^16^,^17^. In our case, the meet/min index takes into account the number of shared genes between two microRNAs, divided by the minimum number of regulated genes between the microRNAs. This metric takes its value between 0 and 1, where 1 implies the exact same targets, whereas 0 implies no common target. The network thus constructed contained 555 nodes and 153,735 weighted edges with a density of 1, meaning that all nodes in the network are interconnected with different strengths.

Many algorithms have been implemented in systems biology to analyse weighted graphs, notably in protein-protein interaction (PPI) networks. Some algorithms are based on maximal cliques finding and ranking. Although algorithms based on this method are NP-hard, it is not a problem in PPI networks due to their sparse properties^18^. However these methods are not suited to our network which is highly dense. As a consequence, and in spite of information loss, the weighted graph was simplified into a binary graph by defining a meet/min threshold through a “multiple-thresholds-approach”^19^,^20^, which consists of the comparison of different thresholded networks.

To define an appropriate threshold, we analysed changes in the network properties for different meet/min thresholds. Density and clustering coefficients are common properties used in this sense^21^. The density measures the number of edges compared to the number of awaited edges if every node were connected in the network. In addition to the number of edges and connected nodes in the graphs, we also considered different centrality measurements such as betweenness and degree centrality^22^. Our aim was to keep the maximum number of connected nodes in the graph but still be able to analyse it.

**Figure 2:**
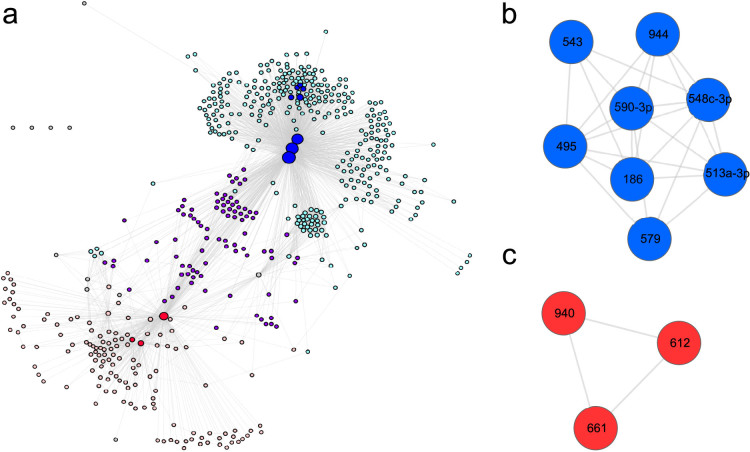
DIANA-microT network at meet/min 0.5. (a)The network can be divided into two parts (pink and cyan) linked by a few common microRNAs in the middle (purple). In cyan are the nodes that are connected to at least one microRNA of the assorted club 1; in pink are the nodes connected to the assorted club 2; and in purple are the nodes connected to both groups. The nodes not directly connected to the assorted clubs are in grey. Four nodes remained isolated from the entire graph; they are shown in the top left part. Node size is proportional to the node degree. (b) Assorted club 1 has a density of 0.8. It comprises miR-495, miR-548c-3p, miR-590-3p, miR-186, miR-579, miR-513a-3p, miR-543 and miR-944. (c) The assorted club 2 has a density of 1 and is composed of miR-661, miR-612 and miR-940.

Every node is connected to at least one neighbour in the graph until meet/min reaches 0.48, at which point the number of edges decreases sharply (Supplementary Figure S1d). At meet/min 0.4, the density is close to 0 (Supplementary Figure S1e), meaning that the graph is sparse (scattered nodes that are not highly connected to each other), in contrast to the weighted one. The global clustering coefficient gives the degree to which nodes in a graph tend to form clusters (or to put it more simply: the number of “triangles” in the network). The clustering coefficient reaches its minimum near meet/min 0.5 (Supplementary Figure S1e). Finally, centrality measurements evaluate the centrality of each node within the network. Betweenness centrality gives for a given node the number of shortest paths from all pairs of nodes that pass through this node, whereas degree centrality gives information on the degree of this node, that is, the number of edges linked to the node. Node-level centrality measurements can additionally be averaged into single graph-level scores. In our case, the two graph-level centrality measurements are high at this 0.5 meet/min threshold (Supplementary Figure S1e and Supplementary Table I). Furthermore, the network contains 555 nodes and 2,911 edges with a density of 0.02 (Figure 2a). Increasing the threshold further leads to networks formed of isolated modules where only microRNA families can be found (let-7, miR-17/miR-93 cluster, etc.) and whose seed sequences are almost identical in each cluster.

To fully appreciate centralities, we compared 3 graphs for 3 different meet/min thresholds (0.25, 0.5, 0.75) to a random network with 555 nodes and 2,911 edges^23^, a scale-free graph iterative construction with 555 nodes^24^ and a human PPI based on the yeast two-hybrid system^25^ (Supplementary Table I). The density of the human interactome graph is much lower than the other graphs except for the meet/min 0.75 and meet/min 1 graphs, where only microRNA families are found (*e.g.*, the let-7 family). The centrality measurements of the meet/min 0.5 graph are in general much higher than every other graph – as is the clustering coefficient – showing an underlying organization of the meet/min 0.5 network based on hubs and closely linked groups of microRNAs. With a diameter (longest of the shortest paths between any two nodes) of 5 and an average path length (average shortest path between all possible pairs of nodes) of 2.5, the meet/min 0.5 graph is a rather compact graph (Figure 2a and Supplementary Table I). We thus set the meet/min threshold to 0.5 as follows: imposing the condition that two microRNAs are connected in the graph only if they share 50% of their targets. Under this condition, the graph shows a slight scale-free behaviour (R^2^ = 0.64), is formed of modules and is dissassortative (Supplementary Figure S2) – a dissassortative network being a network where low degree nodes tend to connect more often to higher degree nodes. It also tends to be a small-world network with high centrality measurements where information is easily transmitted from one node to another, and with central hubs coordinating information. Small-world networks are typical networks where nodes are not all connected to others but are easily reachable through other common nodes. Interestingly, it is at this threshold that we observed these specific characteristics, which are in concordance with our current understanding of biological networks^26^. Although threshold choice is always subjective, we compensated for this arbitrary factor by applying an exploratory statistical analysis to the whole graph.

### Deciphering “assorted clubs” of microRNAs

Barabási and Oltvai defined “modules (or clusters)” as highly interconnected groups of nodes^21^. In our model, a cluster comprises interconnected microRNAs that all share a high number of targets.

To test and decipher the underlying organization based on previously described hubs, we followed a “rich-club strategy”^27^. According to the authors, a rich club can be defined as interconnected hubs in a network with a density of 1 (clique), *i.e.*, a group with a central and influential role. In our case, as the density is high but below 1, we chose to name the groups “assorted clubs” referring to the assortative behaviour of these interconnected and central microRNAs. The analysis of the density formed by the induced subgraph of the *i* first nodes of highest degree (that is, the hubs sorted by their degree) reveals the presence of two assorted clubs (Supplementary Figure S3). At 11 hubs, the first assorted club (assorted club 1) is formed by 8 microRNAs with density of 0.8 and is further emphasised in blue in Figure 2a and b. The second assorted club (assorted club 2) is formed by 3 microRNAs (shown in red in Figure 2a and c) with a density of 1. Knowing that the graph global density is 0.02, the high density values highlight the close connectivity between the different microRNAs and, more interestingly, the high number of shared targets between all of them. Their close connections further reinforce the idea of a common co-regulated biological process between the different microRNAs. As the 12^th^ microRNA is neither connected to the first assorted club nor to the second, we decided to define two clubs with the first 11 degree hubs of the network (Figures 2b and 2c). The two clubs are regrouped into one single network with the 48^th^ microRNA (Supplementary Figure S3).

Despite the normalization imposed by the meet/min formulae, there is still a correlation between the number of potential targets of a microRNA and its number of neighbours in the network (Supplementary Figure S4). Thus, the hubs are not only the microRNAs that are highly connected to other microRNAs but also those with the highest number of predicted targets. Interestingly, most of the hubs also have a high node-level betweenness centrality value (ranging from 0.40 to 0.67). Seven out of the 11 hubs presented here can also be found within the 13 first betweenness centrality sorted nodes. These 7 microRNAs (the 3 microRNAs from the assorted club 2 and 4 from the assorted club 1, namely miR-495, miR-548c-3p, miR-590-3p and miR-186) also seem to be placed at key central positions in the network, defining two separate zones (Figure 2a). The other 4 microRNAs are the remaining members of the assorted club 1. They are, however, more offset on the graph, explaining their lower betweenness centrality values.

To further visualise the structure of the graph organised around the central hubs, we color-coded in cyan the microRNAs linked to at least one of the members of the assorted club 1, in pink the neighbours of at least one of the members of club 2, and in purple the microRNAs connected to at least one member of each cluster (Figure 2a). With this colour scheme, we clearly see that there are three parts structured around the two assorted clubs. The purple part delineates a trench between the two clubs (intermediate zone) that are central to the two extreme zones (cyan and pink). We thus named the two extreme zones as the “sphere of influence” of the assorted clubs. This general organization explains the high graph-level centrality measurements that we observe across the network.

### Assorted club 1

The assorted club 1 is composed of 8 microRNAs (Supplementary Table II), including the microRNA with the highest degree in the graph (miR-495). The latter is connected to 72% of the miRNome – hereby defined as the 555 microRNAs of DIANA-microT v3. miR-495 is also predicted to target 6,626 different genes and has 13,900 different target sites. On average, the microRNAs of this group target approximately 5,000 genes. As many as 5,276 genes are shared by at least 4 microRNAs (50%) of the cluster, and 540 genes are shared by all 8 microRNAs. Within this club, only miR-495 and miR-543 are clustered on the genome. They are both localised on chromosome 14 and separated by approximately 1,500 base pairs (Supplementary Figure S5).

As this cluster is composed of hubs of the highest degree in the network and because there is a correlation between the number of targets and the number of edges for a microRNA, one would expect this group to have low specificity. This can be explained by the fact that microRNA hubs may have to regulate a large number of genes at the same time. Indeed, we can suppose that the more genes a group of microRNAs has to regulate, the less specificity it will have – as other groups regulating a part of those genes would bring redundancy. As such, within the 540 genes shared by all 8 microRNAs, 17% of shared genes of this cluster are gene hubs. This represents 47% of all protein hubs (Supplementary Figure S6a - Fisher exact test *P*-value = 10^−90^).

To further interpret the role of microRNA clusters, we looked at the enrichment of gene ontology (GO)^28^ for the coregulated genes of each cluster. A Benjamini-Hochberg (BH) correction was used to account for multiple testing hypothesis correction^29^, even though we did not consider the corrected *P*-values as pure decision-making values (see the Methods section for a brief discussion). Only the genes that were shared by at least 50% of all members of the clusters were used in this analysis (4 microRNAs). Using the package TopGO^30^ to calculate the GO enrichment, we found enrichment in mRNA processing, transcription and gene expression on the biological process (BP) level of GO (Table 1: assorted club 1), for which BH corrected *P*-values (*P*_BH_) ranged from 10^−5^ to 10^−8^. When considering less generic annotations, we found enrichment for protein modification (*P*_BH_ = 3 × 10^−4^), endosomal transport (*P*_BH_ = 5 × 10^−4^) and regulation of locomotion (*P*_BH_ = 10^−2^). Consistent with these findings, “nucleus” was found as the localization of a significantly high number of genes targeted by the microRNAs (*P*_BH_ = 1.6 × 10^−7^) (Supplementary Table III. Cellular Component). Accordingly, metal ion binding (*P*_BH_ = 6.3 × 10^−10^) was found in the molecular function (MF) category (Supplementary Table III. Molecular Function). Although they are very general annotations, the results correlate with DNA/RNA binding and mRNA processing, showing that the cluster seems to regulate transcription regulators.

Unfortunately, a literature review of the 8 microRNAs reveals little information on their cooperative behaviour and their role in the regulation of transcription factors. However, miR-186 and miR-543 are both cited by different studies in cellular aging^31^,^32^, demonstrating the possibility of their coaction. miR-495 and miR-543 were both identified – with other microRNAs – as actors in the epithelial to mesenchymal transition (EMT)^33^. miR-495 is known to have an effect on cell differentiation and proliferation^34^,^35^,^36^ and is also known to be a tumour suppressor^37^. Although miR-186 is known to have an effect on a proapoptotic purinergic receptor^38^, it is not known whether this microRNA has a direct role on apoptosis. Similarly, miR-590-3p has a role in neuronal death^39^, whereas miR-513a-3p is known to be involved in the immune system response mediated by interferon gamma (IFN-γ)^40^. Finally, miR-548c-3p is involved in the DNA repair process by acting on TOP2A translation^41^. Based on existing knowledge of the biological role of the microRNAs in question, it is difficult to draw conclusions about their complementary behaviour.

Nonetheless, the positions of the members of this assorted club are central to the graph, especially for miR-548c-3p, miR-590-3p, and miR-495 (Figure 2b). By their neighbourhood positioning, they clearly define what we have called a “sphere of influence” represented in cyan in Figure 2a. To understand the biological role of this sphere, we also looked at the GO enrichment using the genes that were shared by 25% of the 315 microRNAs (Table 1: sphere of influence club I and Supplementary Table IV). As most of the coregulated genes of the assorted club and its sphere of influence are shared (Supplementary Figure S6c), one could *a priori* anticipate an enrichment correlation between the two. The sphere appears to be involved not only in transcription regulation – just as the assorted club is itself – but also in development and differentiation (*P*_BH_ ranging from 10^−4^ to 10^−7^). This further demonstrates how the hubs influence the other microRNAs around them. By further restricting the genes used for the enrichment calculation to target genes shared by at least 50% of the 315 microRNAs, we saw a clear focus of the ontology on “nervous system development” (Supplementary Table V). This statement can be explained by the protein hubs introduced earlier. The ontology enrichment of the 193 protein hubs from DIANA-microT shows the same focus on “nervous system development” (Supplementary Table VI). As the limitation imposed for the second enrichment calculation on the sphere (genes shared by 50% of the microRNAs) includes many of the protein hubs (Supplementary Figure S6e), it biases the result of the enrichment toward nervous development processes.

### Assorted club 2

Assorted club 2 is composed of 3 microRNAs (Supplementary Table IV), namely miR-940, miR-661 and miR-612. On average, each microRNA of this cluster is predicted to target 5,254 genes. A total of 4,596 genes are predicted to be regulated by at least 2 microRNAs of the cluster, defining the consensus set of genes used for the GO enrichment. The three microRNAs are localised on three different chromosomes. miR-940 is located on chromosome 16, miR-661 on chromosome 8, and miR-612 on chromosome 11 (Supplementary Figure S5). This second assorted club should exhibit more specificity than the first, as the microRNAs target fewer mRNAs on average than the assorted club 1 microRNAs. Within the 1,830 genes that are shared by all three microRNAs, approximately 6.5% are protein hubs. This represents more than 61% of the DIANA-microT protein hubs (Supplementary Figure S6b - Fisher exact test *P*-value = 10^−71^). Fifty-two protein hubs are shared between the assorted club 1 and this club.

“Small GTPase-mediated signal transduction” was the most enriched GO term at the level of BP (*P*_BH_ = 5 × 10^−3^), followed by terms involved in cell communication and signalling (*P*_BH_ < 0.05) (Table 2: assorted club 2). A significant number of proteins targeted by this cluster are localised in the membrane (cell membrane, organelle membrane, plasma membrane, etc.) and cell junction (*P*_BH_ < 10^−5^) (Supplementary Table VII: Cellular Component). Finally, we found enrichment in phospholipid binding (BH corrected *P*-value = 0.036) when looking at molecular functions (Supplementary Table VII: Molecular Function). No other term passed our statistical criteria, even though small GTPase regulation and binding (“Ras GTPase binding”, “Rho guanyl-nucleotide exchange factor activity”) were also found in the list at less significant *P*-values, confirming the first enrichment described above. Following this clear enrichment in small GTPase signalling, we also found enrichment in cell organization and cellular development, but with higher corrected and uncorrected *P*-values (< 0.001 and < 0.3, respectively). Overall, there was a consistency in enriched GO terms around GTPase signalling, even though *P*-values were not strikingly significant.

**Figure 3:**
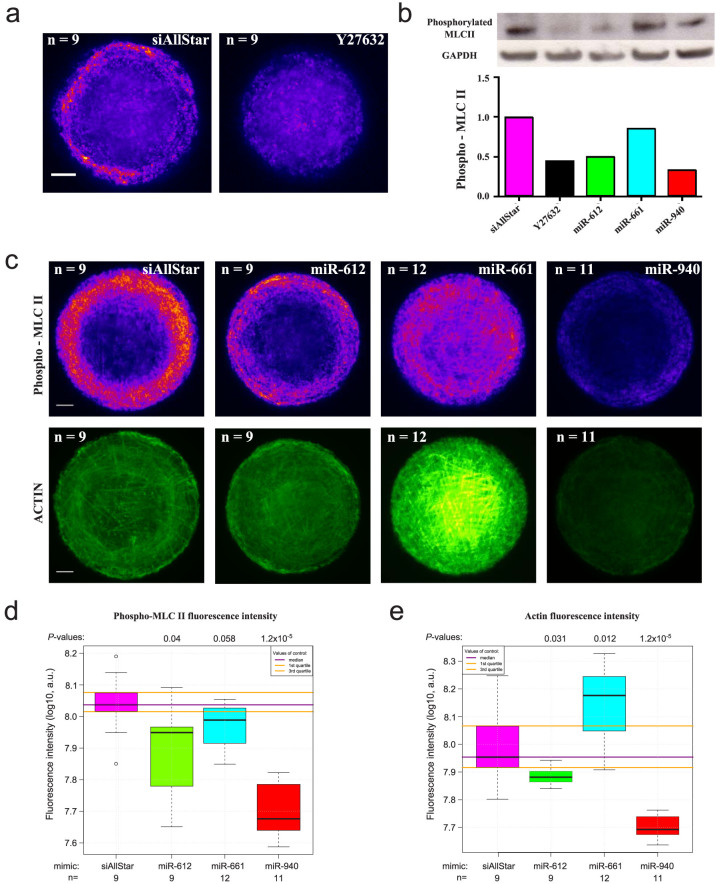
Involvement of miR-661, miR-612 and miR-940 in small GTPase signalling. (a) Immunostaining of phosphomyosin II and actin filaments of RPE1 cells plated on 500 µm^2^ circular fibronectin patterns. RPE1 cells were transfected with siRNA-AllStars (siAllStars, negative control) and Y27632 (ROCK inhibitor). They were immunolabeled for phosphomyosin II. Nine different images for each condition were taken, aligned and projected into a single image by using the median value of all images for each pixel (Median Z projection of ImageJ). Rescaled with the same conditions, the images were color-coded with the “fire” look-up table to highlight intensity variations. Scale bar, 5 µm. (b) Western blot of phosphomyosin II. RPE1 cells were lysed and supplemented with protease inhibitor. A total of 10 µg of proteins were deposited and hybridised to MLCII antibodies. GAPDH was used as a loading control. The bar plot shows the GAPDH-normalised signal rescaled to siAllStars. (c) Immunostaining of phosphomyosin II and actin filaments. RPE1 cells were transfected with miR-612, miR-661 or miR-940 mimics and immunolabeled for phosphomyosin II and actin fibres on 1000 µm^2^ circular fibronectin patterns. For myosin and actin images, 9 to 12 images were taken, aligned and projected into a single image. They were color-coded with the “fire” and “green hot” look-up table to highlight intensity variations for myosin and actin staining, respectively. Scale bar, 5 µm. (d) Log10 of phosphorylated myosin II fluorescence intensity. The integrated fluorescence intensity of myosin was calculated from single images after cell segmentation for each condition. *P*-values were calculated using the non-parametric two-sided Mann-Whitney test and the number of observations (n) for this calculation. a.u.: arbitrary units. (e) Log10 of actin fluorescence intensity. The integrated fluorescence intensity of actin filaments was calculated from single images after cell segmentation for each condition. *P*-values are calculated using the non-parametric two sided Mann-Whitney test and the number of observations (n) for this calculation. a.u.: arbitrary units.

To confirm the prediction regarding this cluster, we looked at the localization and quantified the level of phosphorylated myosin light chain II (MLCII) in Retinal Pigment Epithelial (RPE1) cells using phospho-myosin light chain II antibodies. MLCII is a substrate of Rho-associated protein kinases (ROCK) but is also an ideal readout to monitor small GTPase signalling, as it is the end product of this signalling cascade^42^. Additionally, the cells were plated on micropatterned fibronectin to normalise their shape and actin cytoskeleton architecture, which was also monitored via phalloidin distribution^43^. Figure 3a shows RPE1 cells treated by siRNA-AllStars (negative control) and Y27632, a chemical inhibitor of ROCK used as a positive control, on 500 µm^2^ circular fibronectine patterns. A decrease in the global phosphorylation of MLCII was observed with Y27632. However, as the patterns were small compared to the size of RPE1 cells, RPE1 cells were constricted, which involved less cellular contraction. 1000 µm^2^ circular fibronectine patterns were used in the following experiments so that cells had fewer restrictions and also so that a generally higher level of phosphorylation could be observed. The quantification of MLCII phosphorylation by Western blot on unrestricted cells showed a decrease in phosphorylation with each human microRNA mimic in comparison to the siAllStars negative control (Figure 3b), which confirmed the involvement of the three microRNAs in small GTPase signalling. Furthermore, Figure 3c shows that upon transfection of miR-612 and miR-940 mimics, the cells were relaxed and exhibited the same behaviour as the cells treated with Y27632. Their actin filaments were disorganised, with an absence of stress fibres and transverse arcs compared to cells treated with siRNA-AllStars (Figure 3c). In contrast, the miR-661 mimic revealed a higher number of myosin-decorated stress fibres and highly contracted cells with dense fibres (Figure 3c). However, we clearly observed that miR-661 induced a spatial reorganization of MLCII from the border of the cells to the entire cell surface. With an image-based phosphorylation quantification, we observed that the overall phosphorylation level of MLCII was only significantly reduced with the overexpression of miR-612 and miR-940 (*P*-values = 0.04 and 1.2 × 10^−5^, respectively) (Figure 3d). Finally, an increase in actin filament staining was observed in RPE1 cells treated with miR-661 (*P*-value = 0.012, Figure 3e). In contrast, there was a decrease in actin staining following miR-612 and miR-940 treatment (*P*-values = 0.031 and 1.2 × 10^−5^, respectively).

**Figure 4:**
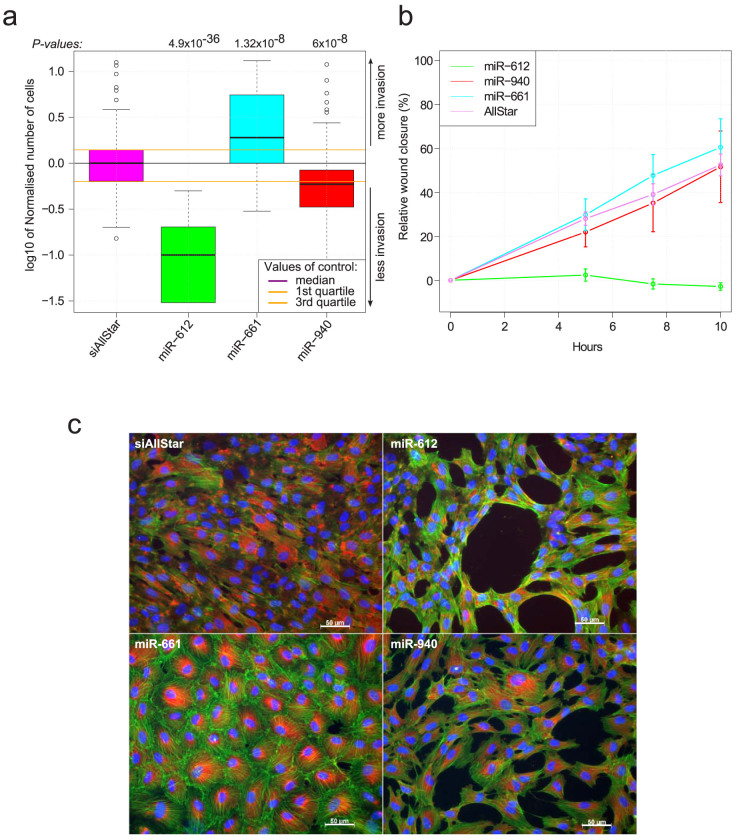
Effect of miR-612, miR-661 and miR-940 on RPE1 migration and proliferation. (a) Motility graph: Normalised number of cells for the transwell assay. RPE1 cells were independently transfected with mimics of miR-661, miR-612 and miR-940. The number of cells that passed through the 5 µm holes after 18 hours were counted. Four independent experiments were conducted. The cell number was normalised based on the negative control cells, and all four experiments were pooled. *P*-values were calculated using the non-parametric two sided Mann-Whitney test. (b) Wound healing assay. Relative wound closure after a scratch was made in confluent cells transfected by mimics of the three microRNAs. The experiment was conducted on 10 hours with images taken at t_0_, t_0_+5h, t_0_+7.5h, and t_0_+10h. Each condition was present in triplicates. (c) Vinculin and phalloïdin immunostaining images of the cells transfected by mimics of the three microRNAs. The images were taken at the AxioImager®.

A transwell assay and a wound healing assay were carried out to investigate the influence of the three microRNAs on the cytoskeleton dynamics and the ability of the cells to modify their shape and migrate. The aim of the transwell assay was to capture the dynamics of the cell cytoskeleton and the ability of cells to migrate through holes whereas the wound healing assay determined the ability of the cells to divide and migrate. miR-661 clearly produced an increase in the number of cells that went through the transwell membrane compared to the control (Figure 4a, *P*-value = 1.3 × 10^−8^, Mann-Whitney test). In the same manner, miR-661 allowed the cells to close the wound faster compared to the control (Figure 4b) but also more constricted cells with increased actin staining (Figure 4c). These two results show that miR-661 greatly enhances cell motility and division when overexpressed. Conversely, miR-612 produced a significant decrease in the number of cells crossing the transwell membrane (*P*-value = 4.9 × 10^−36^, Mann-Whitney test) and completely blocked the closure of the wound. The microRNA also induces changes in the general shape of the cells and their interaction with each other. Indeed, the cells are less constricted and form a hollow network resembling epithelium surface (Figure 4c). In the same way, miR-940 also produced a decrease in the number of cells (*P*-value = 6 × 10^−8^, Mann-Whitney test) but to a lesser extent, and exhibited no clear difference overall on the wound healing assay compared to the control. However, the impact of the microRNA overexpression on the shape of the cells is still highly visible and follows the same trend as miR-612 with, again, a less marked phenotype (Figure 4c). These results strongly support our ontology prediction with respect to the assorted club 2 and our previous observations from the micropatterned assay where strong effects on cell motility and the cytoskeleton organization was observed.

To assess the regulation power of the network microRNA, we then colour-coded the network according to microRNA expression in 8 different normal tissues (notably breast, colorectal mucosa, lung, prostate, blood, prefrontal cortex, liver and muscle). Interestingly, miR-940 is moderately to highly expressed (pink to red node) in 7 tissues out of 8 whereas miR-661 and miR-612 both show little or no expression (white node) in the same tissues. On the other hand, only miR-612 is expressed in normal colorectal mucosa (Supplementary Figure S7 and S8). The three microRNAs seem rarely co-expressed in the different tested tissues. This is consistent with the phenotypic outputs that we observed after ectopic overexpression of these microRNAs in RPE1 cells.

**Figure 5:**
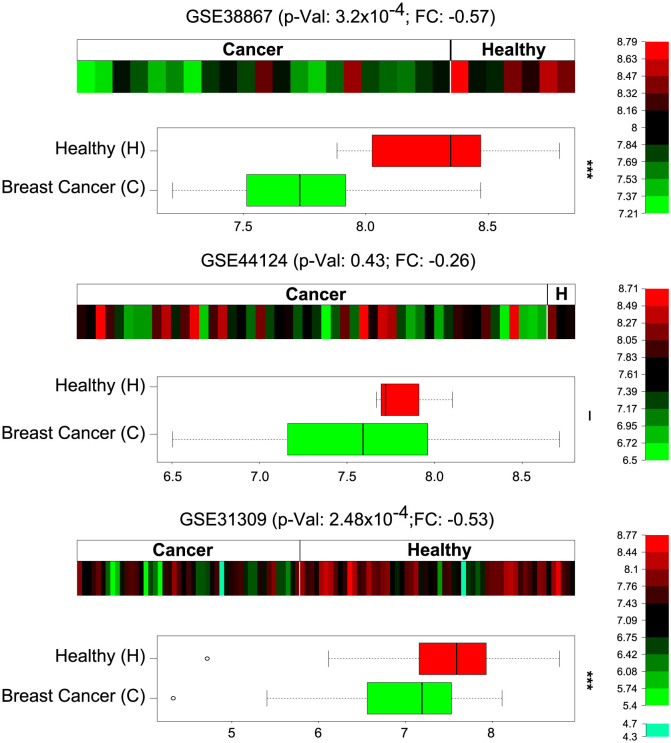
Relative expression of miR-940 in breast cancer. Expression of miR-940 on three datasets of human breast cancer taken from GEO (GSE38867, GSE44124 and GSE31309). The expression of the microRNA is consistently downregulated in breast cancer tissues on the three experiments. On two out of three microarray sets, miR-940 is differentially expressed with high significance based on limma analysis (p-Value < 0.001).

Differential expressions of the three microRNAs between healthy and cancerous tissues were then carried out on Gene Expression Omnibus (GEO) data. Interestingly, miR-940 was found consistently downregulated in breast cancer (log fold change of −0.26 on GSE44124, -0.53 on GSE31309, and -0.57 on GSE38867). Moreover, two out of the three datasets tested showed a statistically very significant differential expression (Figure 5). These data are consistent with our previous results on the role of miR-940 in cell dynamics and might shed light on a probable role of this microRNA in breast cancer progression.

Just as with assorted club 1, the members of assorted club 2 also define their own sphere of influence. This sphere is composed of 129 microRNAs (pink in Figure 2a). With a limitation to the 4,208 genes shared by 25% of the sphere microRNAs (at least 33 microRNAs), there is enrichment mostly for “signalization” but also for “nervous system development”. The corrected *P*-values range from 10^−6^ to 10^−14^ (Table 2: sphere of influence Club 2 and Supplementary Table VIII). The annotations again follow the same trend as the enrichment of the assorted club 2 due to the high number of shared targets (Supplementary Figure S6d). With genes shared by at least 50% of the 129 microRNAs, a higher bias toward nervous system development is observed (Supplementary Table IX). In both cases, the enrichment for brain development can be explained by the protein hubs shared by the microRNAs of the sphere (Supplementary Figure S6f).

The transitory zone of influence (purple on Figure 2) is not highly enriched for any particular process (Supplementary Table X and XI). Indeed, the enrichment of the 3,011 genes shared by at least 25% of the transitory zone (23 microRNAs on 89) is enriched mainly for “transport” with BH corrected *P*-values ranging from 10^−1^ to 10^−4^ (9 first annotations in BP). For genes shared by at least 50% the microRNAs of this zone, no enrichment could be found. So, in general and reassuringly, no clear enrichment is found for the group of microRNAs that are connected to both assorted clubs.

### Robustness of the approach

To assess the robustness of our approach, we compared the results obtained using DIANA-microT v3 to those from TargetScan v6.2 (June 2012)^44^, a prediction tool also based on seed sequence analysis. We chose to limit this study to the non-conserved version of the TargetScan algorithm, as no real proof indicates that targets which are conserved across species are more accurate than non-conserved targets^45^, and also to reduce the number of false negative prediction. Due to the differences in the prediction algorithms, the two databases predict different microRNA targets. These differences in target prediction are further illustrated in Supplementary Figure S9, where we observe that the coverage of microRNA targets between the two databases is – on average – only approximately 60% (meet/min, Supplementary Figure S9). The coverage is slightly higher when considering only the members of the assorted clubs but is still below 70%. The most covered microRNA is miR-513a-3p, with a meet/min value of 84%, whereas the least covered microRNA is miR-543, with a meet/min value of 59% (Supplementary Figure S9a).

Despite the differences in target prediction, the TargetScan network built with the same process described above has a similar two-part structure to that obtained using DIANA-microT (Supplementary Figure S10). The two spheres of influence seen in the DIANA-microT network are also present in the TargetScan network. We also see that the two spheres of influence are likewise organised around a small number of central nodes, comprising the assorted clubs from DIANA-microT. The different properties of the network (clustering coefficient, centrality measurements, number of connected nodes, etc.) are almost identical to those of DIANA-microT network (Supplementary Figure S11). One major difference is the fact that the hubs from the network are different, and as such, the assorted club 2 from the DIANA-microT graph does not come out as an assorted club in TargetScan. However, even though they are no longer hubs, they are still very central to the network and globally still define two spheres of influence (Supplementary Figure S10). The intermediate zone is less obvious, even though the microRNAs from this zone are still mostly located between the two spheres. Despite the fact that we observe three-fold more microRNAs in the TargetScan network, 5 out of the 7 hubs with high betweenness centrality previously described in the DIANA-microT network are found within the 30 first betweenness centrality sorted nodes. The 5 microRNAs are, in decreasing order of centrality: miR-548c-3p, miR-590-3p, miR-661, miR-186 and miR-940. Lastly, 7 hubs from the 11 hubs discovered by DIANA-microT are retrieved within the 40 degree sorted nodes on the TargetScan network (in decreasing order by degree: miR-548c-3p, miR-590-3p, miR-579, miR-186, miR-513a-3p, miR-661, miR-495 and lastly miR-940).

The 11 hubs identified by DIANA-microT were compared with the two prediction algorithms. Interestingly, the two assorted clubs are connected with density profiles that are almost identical in both networks (Supplementary Figure S12a and b). This proves that the connections between the microRNAs are robust across database changes. A major difference is that the two clubs are now connected in the TargetScan network. This connection is made *via* miR-548c-3p (Supplementary Figure S12c). Importantly, when looking at gene ontology enrichment for the two assorted clubs with TargetScan predictions, the results are equivalent to DIANA-microT target ontology enrichment (Supplementary Tables XII and XIII). Surprisingly, even more significant *P*-values are observed for the targets predicted by TargetScan for the assorted club 2 (typically by one order of magnitude), again with a focus on GTPase signalling.

To conclude, even though the hubs are not the same between different target prediction databases and despite all the differences in target prediction, the relations (target sharing and, therefore, connections) between the different microRNAs remain consistent, as do the ontology prediction and the general shape of the networks.

## Discussion

MicroRNAs are crucial entities that regulate diverse biological processes in cells by targeting many different mRNAs. Furthermore, many genes are targeted by at least a few microRNAs. Under these two assumptions, systems biology seems to be the method of choice to characterise the complementary role of microRNAs. Here, we present a way to infer microRNA networks, taking into account target similarities based on the principle that if two microRNAs share similar targets, they may coregulate similar pathways. Tsang *et al*. already suggested that microRNA cotargeting is prevalent in the cell and considered microRNA families in their study^46^, where they hypothesised that different microRNAs targeting the same genes would imply a wider range of target-level modulation. In fact, two microRNAs can also be functionally related if they regulate different genes that reside in the same pathways, a question that was addressed by Xu *et al.*^6^ when they developed an approach for microRNA networks based not only on target sharing but also on similarities in GO biological processes. In our case, we sought to obtain information on the processes that the group might coregulate rather than to use ontology as a tool to infer networks. More importantly, we focused our analysis on the role of hub microRNAs, i.e., microRNAs that are more connected than others due to their high number of predicted targets.

Although it is known that *in silico* predictions have a high percentage of false positives, we decided to keep every available prediction in our analysis. With the sensitivity of microRNA target prediction algorithms being approximately 66%^47^, some studies rely solely on experimentally validated microRNA-gene interactions^48^. However, even though these data may be considered more robust, they are still very limited. For example, only one target is validated for miR-612 in the latest miRTarBase release v4.5^49^, and none of those in miRecords v4 are validated^50^. Not only do the predictions have a high false positive rate, but the coverage between different algorithms is also low (approximately 60% between TargetScan v6.2 and DIANA-microT v3: Supplementary Figure S9). Regardless of the aforementioned issues, the networks built independently with the two algorithms gave almost equivalent results, both in terms of network architecture and in terms of hypotheses for the biological implications of the two groups of interconnected hubs.

The interconnected hubs defined two spheres of microRNAs separated by an intermediate zone. A high correlation between the enrichment of the assorted clubs and their respective spheres could be observed, hence the name “sphere of influence”. An important idea in our analysis is the notion of global exploration, meaning that even though the two spheres are mostly involved in their own respective pathways, some microRNAs of the two zones might have little or no involvement in the corresponding pathways. Keeping in mind that we restricted our analysis to a global view of microRNA-mediated biological regulation, we note the link between the structure of the graph (two subnetworks with central hubs) and the biological functions shared by many (but not all) microRNAs in each sphere of influence.

The second central hub group, named “assorted club 2”, was composed of miR-612, miR-661 and miR-940. Very little has been reported on the functional role of these microRNAs in human cells. The analysis of GO among gene targets of the 3 microRNAs was enriched in terms related to small GTPase-mediated signal transduction and, as a consequence, may show an involvement of the 3 microRNAs on the cytoskeleton and affect cell motility. We performed functional validation experiments and confirmed that mimics of each of these microRNAs were acting on the cytoskeleton through phosphorylation of myosin II, a key molecular step in cytoskeleton control. However, the introduction of the microRNAs into RPE1 cells induced different phenotypic outcomes. Strikingly, the ectopic expression of miR-661 strongly modified the spatial phosphorylation of myosin II, while in contrast, overexpression of either miR-612 or miR-940 inhibited myosin II phosphorylation (Figure 3). This antagonistic phenotypic outcome was further confirmed by invasion experiments (Figure 4). Together, these experimental validations confirmed the involvement of the assorted club 2 in the regulation of small GTPase signalling, the actin cytoskeleton, cell motility and cell invasion. Because the three microRNAs lead to different phenotypic effects, it might be not surprising that these microRNAs are not expressed at the same time in a tissue. Confirming this statement, the three miRNAs were not found co-expressed in breast, prostate, colorectal mucosa, lung, blood, prefrontal cortex, liver and muscle normal tissues. Therefore, it would have been difficult to infer this network relying on the expression level of microRNAs. These data further emphasise the relevance of the target-based microRNA networks that we have inferred.

To our knowledge, miR-940 had never been reported as differentially express in breast cancer. This absence might be explained by the fact that miR-940 is never one of the most differentially expressed microRNA in these datasets even though its expression trend is consistent. Here, we demonstrated for the first time its capacity to modulate cell cytoskeleton and reduce RPE1 cell migration and invasion and showed that its expression is reduced in breast cancer (Figure 5). Also in agreement with our results, it was recently shown that miR-612 exerts an inhibitory effect on hepatocellular carcinoma, proliferation, migration, invasion, and metastasis. Moreover, miR-612 appears to be involved in both the initial and final steps of the metastatic cascade by suppressing local invasion and distant colonization^51^. Similarly, our results appear to be in agreement with reports from Vetter *et al.*, who have shown that miR-661 contributes to breast cancer cell invasion through the targeting of Nectin-1 and StarD10^52^. Furthermore, we demonstrated here that miR-612 and miR-661 also regulate cell motility via opposite effects on myosin II phosphorylation.

In the near future, we will further investigate the mechanism of action of miR-661 with regard to the p53 status of the cells, as it was recently reported that miR-661 may either suppress or promote cancer aggressiveness, depending on the p53 status^53^.

## Methods

### Target prediction datasets

The flat file of DIANA-microT version 3.0 (July 2009)^13^ was downloaded from the web site http://diana.cslab.ece.ntua.gr/microT/. The database consists of genome-wide computationally predicted associations between microRNAs and their predicted targets in ensemble id format. Only *Homo sapiens* gene-microRNA information was considered. Scores and multiple binding sites were not taken into account (miTG score > 0), so that the lowest possible level of false negative prediction was considered. Under this restriction, there are 555 microRNAs that are predicted to regulate 18,986 different genes. The DIANA-microT dataset comprised our main dataset for network building.

To compare the network outcome with that of another prediction algorithm, TargetScan v6.2 non-conserved^44^ was downloaded from the web site http://www.targetscan.org/vert_61/ (June 2012) (Date of access: 15/06/2013). Again, only *Homo sapiens* gene–microRNA information were considered, which led to 1,531 microRNAs regulating 18,366 genes. No other criterion of selection was used.

### Network building

Based on the idea that microRNAs that share the same targets might act in the same pathways, a microRNA undirected weighted graph G = (N, E) was built, where each node N represents a microRNA and each edge E represents the percentage of shared targets. To define the percentage of shared targets, the meet/min index (or Simpson index) was used. The metric is highlighted in ref. 16 and can be simply defined as: 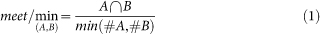
where A and B represent the sets of genes regulated by microRNA A and microRNA B, respectively, and # stands for the number of targets regulated by the corresponding microRNA. The network built upon this metric is a densely connected network with 555 nodes and 153,735 weighted edges.

To analyse this dense weighted network, it was further simplified into a binary network by defining a meet/min threshold under which edges between microRNAs in the network were deleted. To do so, different graph properties were calculated and compared for different meet/min thresholds. We calculated the clustering coefficient, two different centrality measurements (degree and betweenness centrality), the density, and the assortativity coefficient^54^. We defined the meet/min threshold as 0.5. The final network comprised 555 nodes and 2,911 edges.

These microRNA graphs were also compared to 3 different graphs. The first graph was a random graph based on the Erdős-Rényi random graph construction algorithm^23^, where each edge has the same probability of appearance during construction. We considered for this construction 551 nodes and 2,911 edges, as for the meet/min 0.5 network. The second graph was a scale-free graph based on the Barabási-Albert model^24^ considering 551 nodes; at the end of the construction, the network will have scale-free properties as defined by Barabási and Albert. The final graph is a real network of a human protein-protein interaction map based on yeast two-hybrid^25^.

The graphs were built using the package igraph^55^ from the R statistical environment^56^ and visualised using Cytoscape with the unweighted spring embedded layout^57^.

### Assorted club deciphering

First, the hubs of the network were sorted out according to the degree of each node. We then looked at the induced subgraph formed by the i^th^ first sorted hubs (beginning with the first two hubs). Ultimately, 11 hubs were considered, defining two “assorted clubs”^27^: the first comprising 8 microRNAs, and the second comprising 3 microRNAs. The 12^th^ hub was isolated from the 11 first hubs.

### Ontology enrichment calculation for microRNA clusters

The ontology enrichments were calculated using the R package topGO^30^. All levels of gene ontology (GO) were considered: biological process (BP), molecular function (MF) and cellular component (CC). For BP terms, only those annotated for less than 5,000 genes and more than 10 genes were considered. To calculate the enrichment, Ensembl ids were used with DIANA-microT v3 predictions, and Entrez ids were used with TargetScan v6. All genes predicted by the respective algorithms were used as background genes for the two enrichment analyses. The ontology enrichment in terms of biological processes, molecular function and cellular component categories was built on genes that were shared by at least 50% of the group of microRNAs (*e.g.*, for a cluster of 3 microRNAs, a gene must be predicted to be regulated by at least 2 microRNAs to be considered). For the spheres of influence, a lower threshold of 25% was also used to reduce the restriction on the shared genes.

A classic Fisher's exact test was considered for the enrichment analysis. *P*-values were adjusted using the Benjamini and Hochberg correction^29^, and all enriched annotations obtained for each level of GO were used in the multiple testing step (4,109 in BP, 1,277 in CC, and 3,732 in MF with DIANA-microT background genes). The corrected *P*-values were considered significant when < 0.05, although we did not consider this criteria as a strict decision-making threshold. The reader can refer to other publications for a discussion^58^,^59^. Briefly, multiple testing procedures generally suppose that all tested hypotheses are independent. In the case of GO enrichment, the structure of the tree involves dependences among the different annotations, which make the BH correction too stringent in this case. We thus also considered the annotations with less significant corrected *P*-values.

### Cell culture and transfection

Human telomerase-immortalised Retinal Pigmented Epithelial cells (hTERT-RPE1 or RPE1, Takara Bio Inc., Kyoto, Japan) were grown at 37°C and 5% CO_2_ in DMEM/F12 (Invitrogen, Carlsbad, California) supplemented with 10% fetal calf serum, 2 mM glutamine, 100 U/ml penicillin and 100 U/ml streptomycin. Reverse transfection was performed using lipofectamine RNAiMax (Invitrogen, Carlsbad, California) for 48 h. The final concentration of microRNA mimic and siRNA was 20 nM. The microRNA mimics (miR-612, miR-940 and miR-661) were purchased from Thermo Scientific (Waltham, Massachusetts) Dharmacon (miRIDIAN). The siRNA sequence AllStars scramble (Qiagen, Venio, The Netherlands) was used as a negative control, and ROCK inhibitor Y27632 (refY0503: Sigma-Aldrich, St. Louis, Missouri) was used as a positive control. Y27632 was added at 10 µM for the last 24 h.

### Cell lysis, protein extraction, and Western blotting

Protein lysates were prepared in ice-cold RIPA (Thermo Scientific), supplemented with protease cocktail inhibitor (complete mini; Roche, Basel, Switzerland), 1 mM PMSF, 2 mM Na_3_VO_4_, and glycerophosphate. Homogenates were cleared by centrifugation at 15,000 g for 15 min at 4°C. A total of 10 µg of proteins were run on SDS-polyacrylamide gels and blotted onto nitrocellulose membranes. Blots were blocked in 3% BSA (in TBST) for 1 h and then incubated with the primary polyclonal rabbit antibody against phosphomyosin light chain 2 (1:1000; Cell Signaling Technology, Danvers, Massachusetts) in 3% BSA overnight at 4°C. Visualisation was performed using a horseradish peroxidase-conjugated antibody (anti-rabbit; Santa Cruz Biotechnology, Dallas, Texas). For the loading control, a GAPDH rabbit polyclonal antibody was used.

### Micropatterning

Glass coverslips were first spin coated at 3,000 rpm for 30 s with an adhesion promoter (TI Prime; MicroChemicals, Madhya Pradesh, India) and then with 0.5% polystyrene dissolved in toluene. The polystyrene layer was further oxidised with an oxygen plasma treatment (FEMTO; Diener Electronics, Germany) for 10 s at 30 W and incubated with 0.1 mg/ml polylysine polyethylene-glycol (JenKem Technology, Beijing, China) in 10 mM Hepes, pH 7.4, at room temperature for 1 h. Coverslips were then dried by spontaneous dewetting. Polyethylene-glycol–coated slides were placed in contact with an optical mask holding the transparent micropatterns (Toppan Photomasks, Round Rock, Texas) using a home-made vacuum chamber and exposed for 5 min to deep UV light (UVO Cleaner; Jelight Company, Irvine, California). Micropatterned slides were washed once in PBS and finally incubated for 30 min with a solution of 50 µg/ml bovine fibronectine solution (Sigma-Aldrich, St. Louis, Missouri) and 5 µg/ml Alexa Fluor 646– or Alexa Fluor 542–labelled fibrinogen (Invitrogen, Carlsbad, California). Before plating cells, patterned coverslips were washed three times with sterilised PBS. The small circular fibronectine micropattern has a size of 500 µm^2^, and the large circular fibronectine micropattern is 1000 µm^2^.

### Immunostaining

RPE1 cells were transfected with AllStars siRNA or mimics of miR-612, miR-661 or miR-940 at 20 nM for 48 h. The positive control (Y27632) was added to the siRNA AllStars-transfected cells at 10 µM for the last 24 h. The transfected cells were then plated on the micropatterns.

After cell adhesion onto the micropatterns (2 h), RPE1 cells were pre-permeabilized for 15 seconds with 0.1% Triton X-100 in cytoskeleton buffer pH 6.1 and fixed in 4% paraformaldehyde in cytoskeleton buffer for 15 min at room temperature. They were then rinsed twice with PBS and incubated in 0.1 M ammonium chloride in PBS for 10 min. Cells were then blocked with 3% BSA in PBS^Ca2+ Mg2+^ for 30 min.

Images were taken with an upright microscope (BX61; Olympus, Tokyo, Japan). Fluorescent images of myosin and actin staining are maximal projections of different aligned cells acquired with oil immersion objectives at 100× (NA = 1.4) mounted on a piezo ceramic (Physics Instruments, Lederhosen, Germany). Five individual and typically representative images can be found in Supplementary Figure S13. The microscope was controlled with Metamorph software (MDS Analytical Technologies, Toronto, Canada). The images were processed using ImageJ software^60^. To measure the intensity of myosin and actin fluorescence, individual cells were first segmented, and the integrated density of fluorescence was calculated on the segmented cells for both phosphomyosin and actin. The segmentation was performed as follows: a median filter with a radius of 15 pixels was applied on the FITC channel images (phalloidin-stained images), followed by an auto-thresholding using the “Li” method.

For the unrestricted cell images, the cells were transfected in an 8 wells with mimics of the siRNA as previously described. Vinculin and phalloidine staining were used and images were taken at the AxioImager® (ZEISS, Oberkochen).

### Transwell assay

RPE1 cells were seeded in six-well microtiter plates, cultured for one day, and then transfected with mimics of miR-612, miR-661, or miR-940 or with a negative control siRNA (siRNA AllStars) at a final concentration of 20 nM. Twenty-four hours after transfection, cells were trypsinized, re-suspended in culture medium without fetal bovine serum (0% FBS) and counted using a Scepter™ 2.0 (Merck Millipore, Billerica, Massachusetts). A similar cell number was then dispensed on the Transwell® inserts (Corning, polycarbonate membrane, 5.0 µm pore size), and cell migration was induced by the presence of complete culture medium containing serum (10% FBS) in the lower compartments. After 18 hours, cell migration was stopped, and cells were washed (PBS^Ca2+ Mg2+^), fixed (PFA 4%) and permeabilized (100% methanol). For cell counting, cell nuclei were stained with Hoechst 33342, and cells from the deposition side (upper compartment) were removed with a cotton swab. Images of the lower side of the insert were captured (10 different fields of view per insert) with an epifluorescence microscope (Imager Z1 from Zeiss, Oberkochen, Germany) using AxioVision software with a 10-fold objective (Plan-Neofluar 10x/0.30). The quantification of the number of cells that reached the lower side of the insert was performed either semi-automatically using ImageJ software^60^ or manually.

Four independent experiments with varying initial numbers of deposited cells (to better emphasise either the increase or decrease in the number of cells passing through the holes) were conducted. Each condition (miR-612, miR-661, miR-940, and siRNA-AllStars) was represented in triplicate, leading to 30 observations per experiment and per condition (Supplementary Figure S14). To pool the four experiments together, each condition of each experiment was normalised to the median number of cells in the siRNA-AllStars condition (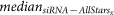), considering all replicates: 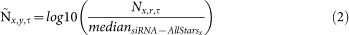
where N is the number of counted cells and Ñ the normalised number of cells for each condition, replicate and experiment; x is the experiment (1, 2, 3 or 4), r, the replicate for each experiment (1, 2 or 3), and, the different conditions (siRNA-AllStars or mimics of miR-612, miR-661 or miR-940). Ñ is thus normalised at 0 for siRNA-AllStars. *P*-values were then calculated using the non-parametric two-sided Mann-Whitney test (Wilcoxon test) available in R statistical software.

### Wound healing assay

RPE1 cells were deposited into a transparent-bottomed 96-well microtiter plate with DMEM medium in presence of 10% FBS and without any antibiotics. After 24 h, the RPE1 cells were transfected either with siAllStars or the 3 microRNAs at 20 nM in triplicates. 48 hours after transfection, 500 µm-large wounds with low width variability were produced in every confluent cell cultures using a wound replicator equipped with 96 pins (V&P Scientific, San Diego, California). The wounds were immediately imaged as well as 5 h, 7.5 h and 10 h after wound formation. No image was taken 2.5 h after wound formation in order to let the cells recover from mechanical and thermal stress resulting from the process of wound formation.

At every time point, the images of the wounds were acquired at 8 exposure times using parallelised holographic microscopy, *i.e.*, an array of 96 Complementary Metal Oxide Semiconductor (CMOS) image sensors (STMicroelectronics, Grenoble, France) placed under the 96-well microtiter plate^61^. Holographic microscopy relies on the digital recording of the diffraction patterns (*i.e.*, holograms) made by the cells under coherent illumination. The 8 images taken at various exposure times for every time point were combined to produce a contrasted, non-saturated image using a High Dynamic Range (HDR) approach. The edges of the wound were automatically detected on the HDR images by a k-means/Markov random field process and a parallel double snake^62^. Results of automated wound segmentations were validated by eye. Evolution of the average width of the wounds was finally normalised in relation to the initial wound width and pooled for every transfection condition.

### Expression data

To assess for microRNA expression in different tissues, samples from 8 different normal tissues were downloaded from GEO. The 8 downloaded datasets comprised GSE19505 (Prefrontal cortex and liver), GSE23527 (Skeletal muscle), GSE24205 (Blood), GSE25508 (Lung), GSE31309 (Breast), GSE34933 (Prostate), and GSE38309 (Colorectal mucosa). The raw data were downloaded and extracted from GEO. Datasets showing negative numbers were adjusted by adding a constant such that the minimum observed value was equal to 1 and then log2 transformed. Finally, a quantile normalization was applied^68^. Cytoscape was used with the DIANA-microT network to visualize microRNA expression levels for each dataset separately. Represented on the networks is the median value of each microRNA expression in control tissues. Min and max observed expression on each dataset were used to define the legend limits with a continuous gradation from white to red. The white limit was set as the median expression on each dataset so that not all nodes would be highlighted. As a consequence, only microRNAs with expression 50% over all miRNA expression(s) in a dataset (moderately to highly expressed on a chip) are coloured. Differences in colour spreading thus reflect the difference in expression score distribution across datasets (Supplementary Figure S15).

For the differential expression analysis, the raw data of the three breast cancer microRNA expression datasets were retrieved on Gene Expression Omnibus (GEO)^63^ under the ID GSE31309, GSE38867 and GSE44124. Only datasets with at least 20 samples were considered. As such, GSE31309 comprises 105 samples with 57 healthy controls. GSE38867 is composed of 28 samples with 7 normal tissues, and finally GSE44124 is built of 53 samples with 3 pools of normal tissue. All data were log2 transformed and quantile normalised^64^ using either affyPLM package^65^ or AgiMicroRna package^66^ in R, depending on the data type. After data normalisation, the package Limma^67^ was used to calculate *P*-values for differential expression of miR-940 in breast cancer tissues against normal tissues. As we investigated only one microRNA per dataset, no correction for multiple testing was applied.

All calculations in this paper were conducted in the statistical environment R^56^. Box-and-whisker plots show the lower and upper quartiles (25–75%) with a line at the median. Whiskers extend to 1.5 times the interquartile range (defined as quartile 75% – quartile 25%). The circles show data outside the whiskers (“outliers”).

## Supplementary Material

Supplementary Information Supplementary information

Supplementary Information Dataset 1

Supplementary Information Dataset 2

Supplementary Information Dataset 3

Supplementary Information Dataset 4

Supplementary Information Dataset 5

Supplementary Information Dataset 6

Supplementary Information Dataset 7

Supplementary Information Dataset 8

Supplementary Information Dataset 9

Supplementary Information Dataset 10

Supplementary Information Dataset 11

Supplementary Information Dataset 12

Supplementary Information Dataset 13

## Figures and Tables

**Table 1:** Gene Ontology enrichment of the assorted club 1 and its sphere of influence (biological process). Only the 20th first terms of each list are represented. In green are represented the terms related to nervous system development, a bias induced by the protein hubs

Assorted club I (5,276 genes shared by 50% of the microRNAs – at least 4 microRNAs/8)					
GO.ID	Term	Annotated	Significant	classicFisher	BH.pval
GO:2000112	regulation of cellular macromolecule biosynthetic process	2817	989	7.50E-12	3.08E-08
GO:0019219	regulation of nucleobase-containing compound metabolic process	3046	1058	2.10E-11	4.31E-08
GO:0031326	regulation of cellular biosynthetic process	2998	1040	5.00E-11	6.85E-08
GO:0010556	regulation of macromolecule biosynthetic process	2877	1000	8.50E-11	8.73E-08
GO:0051252	regulation of RNA metabolic process	2674	935	1.10E-10	9.04E-08
GO:0009889	regulation of biosynthetic process	3026	1045	1.50E-10	1.03E-07
GO:0051171	regulation of nitrogen compound metabolic process	3121	1074	1.90E-10	1.12E-07
GO:0006355	regulation of transcription. DNA-dependent	2602	909	2.80E-10	1.44E-07
GO:0010468	regulation of gene expression	3004	1035	3.60E-10	1.64E-07
GO:2001141	regulation of RNA biosynthetic process	2618	912	5.10E-10	2.10E-07
GO:0006351	transcription. DNA-dependent	2847	980	1.70E-09	6.35E-07
GO:0031323	regulation of cellular metabolic process	3967	1322	9.20E-09	3.15E-06
GO:0034645	cellular macromolecule biosynthetic process	3685	1231	2.40E-08	7.59E-06
GO:0009059	macromolecule biosynthetic process	3756	1251	3.70E-08	1.09E-05
GO:0032774	RNA biosynthetic process	2909	984	9.50E-08	2.60E-05
GO:0019222	regulation of metabolic process	4375	1435	1.40E-07	3.60E-05
GO:0060255	regulation of macromolecule metabolic process	3757	1245	1.50E-07	3.63E-05
GO:0080090	regulation of primary metabolic process	3884	1283	2.00E-07	4.57E-05
GO:0070647	protein modification by small protein conjugation or removal	612	240	2.80E-07	6.06E-05
GO:0044249	cellular biosynthetic process	4466	1453	1.10E-06	2.26E-04

Sphere of influence Club I (4,208 genes shared by 25% of the microRNAs – at least 79 microRNAs/315)					
GO.ID	Term	Annotated	Significant	classicFisher	BH.pval
GO:0007399	nervous system development	1539	472	8.10E-11	3.33E-07
GO:2000112	regulation of cellular macromolecule biosynthetic process	2817	790	7.70E-09	1.58E-05
GO:0010556	regulation of macromolecule biosynthetic process	2877	799	4.20E-08	4.81E-05
GO:0019219	regulation of nucleobase-containing compound metabolic process	3046	840	6.50E-08	4.81E-05
GO:0031323	regulation of cellular metabolic process	3967	1069	7.80E-08	4.81E-05
GO:0048869	cellular developmental process	2496	700	8.10E-08	4.81E-05
GO:0031326	regulation of cellular biosynthetic process	2998	827	8.20E-08	4.81E-05
GO:0009889	regulation of biosynthetic process	3026	832	1.40E-07	6.99E-05
GO:0023052	signaling	4226	1130	1.60E-07	6.99E-05
GO:0035556	intracellular signal transduction	1716	497	1.70E-07	6.99E-05
GO:0030154	cell differentiation	2348	657	3.40E-07	1.20E-04
GO:0007154	cell communication	4338	1154	3.50E-07	1.20E-04
GO:0000902	cell morphogenesis	843	263	4.20E-07	1.33E-04
GO:0006351	transcription. DNA-dependent	2847	782	4.80E-07	1.36E-04
GO:0006355	regulation of transcription. DNA-dependent	2602	720	5.00E-07	1.36E-04
GO:0051252	regulation of RNA metabolic process	2674	738	5.30E-07	1.36E-04
GO:0032502	developmental process	3958	1058	5.90E-07	1.42E-04
GO:0007167	enzyme linked receptor protein signaling pathway	784	246	6.20E-07	1.42E-04
GO:2001141	regulation of RNA biosynthetic process	2618	723	6.60E-07	1.42E-04
GO:0009653	anatomical structure morphogenesis	1827	521	6.90E-07	1.42E-04

**Table 2:** Gene Ontology enrichment of the assorted club 2 and its sphere of influence (biological process). Only the 20th first terms of each list are represented. In green are represented the terms related to nervous system development, a bias induced by the protein hubs

Assorted Club II (4,596 genes shared by 50% of the microRNAs – at least 2 microRNAs/3)					
GO.ID	Term	Annotated	Significant	classicFisher	BH.pval
GO:0007264	small GTPase mediated signal transduction	575	186	2.40E-06	5.67E-03
GO:0007154	cell communication	4338	1148	3.00E-06	5.67E-03
GO:0023051	regulation of signaling	1778	503	5.00E-06	5.67E-03
GO:0009966	regulation of signal transduction	1553	444	6.20E-06	5.67E-03
GO:0007399	nervous system development	1539	440	6.90E-06	5.67E-03
GO:0035556	intracellular signal transduction	1716	484	1.10E-05	6.68E-03
GO:0023052	signaling	4226	1113	1.30E-05	6.68E-03
GO:0048583	regulation of response to stimulus	2027	563	1.30E-05	6.68E-03
GO:0007265	Ras protein signal transduction	353	118	3.40E-05	1.55E-02
GO:0007169	transmembrane receptor protein tyrosine kinase signaling pathway	581	180	5.70E-05	2.34E-02
GO:0016192	vesicle-mediated transport	858	254	6.50E-05	2.43E-02
GO:0007165	signal transduction	3789	994	1.00E-04	3.42E-02
GO:0048011	nerve growth factor receptor signaling pathway	219	77	1.30E-04	4.11E-02
GO:0006897	endocytosis	335	110	1.40E-04	4.11E-02
GO:0010646	regulation of cell communication	1317	371	1.50E-04	4.11E-02
GO:0051056	regulation of small GTPase mediated signal transduction	336	110	1.60E-04	4.11E-02
GO:0035725	sodium ion transmembrane transport	16	11	1.90E-04	4.59E-02
GO:0009653	anatomical structure morphogenesis	1827	499	2.70E-04	6.16E-02
GO:0051179	localization	3782	986	3.00E-04	6.49E-02
GO:0007167	enzyme linked receptor protein signaling pathway	784	229	3.50E-04	7.04E-02

Sphere of Influence Club II (3,605 genes shared by 25% of the microRNAs – at least 33 microRNAs/129)					
GO.ID	Term	Annotated	Significant	classicFisher	BH.pval
GO:0007154	cell communication	4338	1020	1.40E-18	5.75E-15
GO:0023052	signaling	4226	986	1.30E-16	2.67E-13
GO:0007399	nervous system development	1539	406	1.10E-13	1.51E-10
GO:0023051	regulation of signaling	1778	455	5.30E-13	5.44E-10
GO:0007268	synaptic transmission	574	176	1.10E-11	9.04E-09
GO:0035637	multicellular organismal signaling	652	193	3.30E-11	2.26E-08
GO:0007165	signal transduction	3789	860	7.50E-11	4.40E-08
GO:0007264	small GTPase mediated signal transduction	575	173	9.00E-11	4.57E-08
GO:0009966	regulation of signal transduction	1553	394	1.00E-10	4.57E-08
GO:0019226	transmission of nerve impulse	644	189	1.20E-10	4.93E-08
GO:0007265	Ras protein signal transduction	353	117	2.10E-10	7.84E-08
GO:0048666	neuron development	714	204	2.80E-10	9.59E-08
GO:0022008	neurogenesis	1010	271	4.10E-10	1.30E-07
GO:0035556	intracellular signal transduction	1716	425	5.40E-10	1.58E-07
GO:0048856	anatomical structure development	3476	789	8.30E-10	2.27E-07
GO:0030030	cell projection organization	822	226	1.40E-09	3.60E-07
GO:0030182	neuron differentiation	876	238	1.60E-09	3.87E-07
GO:0007409	axonogenesis	479	145	2.00E-09	4.57E-07
GO:0048699	generation of neurons	952	254	2.80E-09	5.96E-07
GO:0048731	system development	3014	691	2.90E-09	5.96E-07
