# Supplementary material for: A statistically inferred microRNA network identifies breast cancer target miR-940 as an actin cytoskeleton regulator
Source: Sci Rep. 2015 Feb 12;5:8336. doi: 10.1038/srep08336 (PMC5389139; doi:10.1038/srep08336)
Supplement: Supplementary Information [file srep08336-s1.pdf]

A statistically inferred microRNA network identifies breast cancer target miR-940 as an actin cytoskeleton regulator

Ricky BHAJUN[1,2,3,α], Laurent GUYON[1,2,3,α], Amandine PITAVAL[1,2,3], Eric SULPICE[1,2,3], Stéphanie COMBE[1,2,3], Patricia OBEID[1,2,3], Vincent HAGUET[1,2,3], Itebeddine GHORBEL[1,2,3], Christian LAJAUNIE[4,5,6] and Xavier GIDROL[1,2,3,*]

[1]Univ. Grenoble Alpes, iRTSV-BGE, F-38000 Grenoble, France

[2]CEA, iRTSV-BGE, F-38000 Grenoble, France

[3]INSERM, BGE, F-38000 Grenoble, France

[4]Center for Computational Biology - CBIO, Mines ParisTech, F-77300 Fontainebleau, France

[5]Institut Curie, F-75248 Paris, France

[6]INSERM, U900, F-75248 Paris, France

[α]These two authors equally contributed to the work

[*]xavier.gidrol@cea.fr

Supplementary Figures

Supplementary Figure S1. DIANA-microT characteristics and network properties of the DIANA-microT network.

Supplementary Figure S2. Characteristics of DIANA-microT meet/min 0.5 graph.

Supplementary Figure S3. Assorted club detection.

Supplementary Figure S4. Number of predicted targets versus the degree of microRNAs.

Supplementary Figure S5. microRNA positions on the genome.

Supplementary Figure S6. Venn diagrams of target coverage between protein hubs, assorted clubs and their respective spheres of influence.

Supplementary Figure S7. DIANA-microT v3 network with microRNA expression of breast, colorectal mucosa, lung and prostate normal tissues.

Supplementary Figure S8. DIANA-microT v3 network with microRNA expression of blood, prefrontal cortex, liver and muscle normal tissues.

Supplementary Figure S9. Target prediction coverage between DIANA-microT v3 and TargetScan Non-Conserved v6.2.

Supplementary Figure S10. TargetScan Non-Conserved network.

Supplementary Figure S11. Network properties of the TargetScan Non-Conserved network.

Supplementary Figure S12. Induced subgraphs and density profiles of the two assorted clubs from DIANA-microT and TargetScan Non-Conserved analysis.

Supplementary Figure S13. Immunofluorescence in individual images of actin and myosin staining on 1000µm² circular fibronectin patterns.

Supplementary Figure S14. Motility graph: number of cells in the transwell assays.

Supplementary Figure S15. Example histograms of microRNAs expression.

Supplementary Figure S1

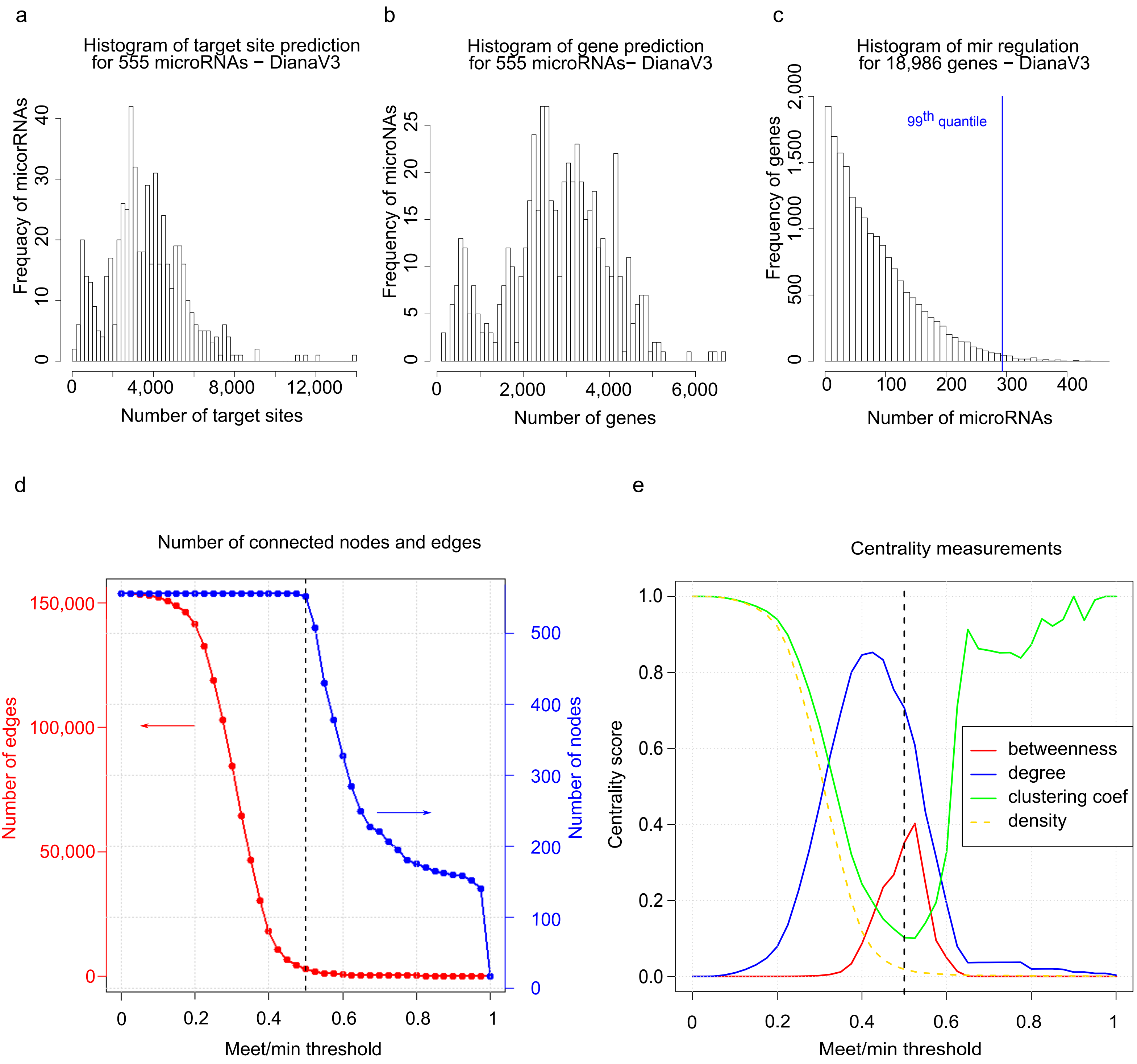

Supplementary Figure S1. DIANA-microT characteristics and network properties of the DIANA-microT network.

a. Histogram of targetsite prediction. One gene can have many targetsites.

b. Histogram of gene prediction.

c. Histogram of microRNA regulation.

d. Number of edges and connected nodes as a function of meet/min thresholds.

e. Density, clustering coefficient, betweenness and degree centrality measurements as a function of meet/min thresholds. The increment of the meet/min threshold is 0.05 from 0 to 1. The 0.5 meet/min threshold results in a high number of connected nodes with a low number of edges (low density) and high centrality measurements (betweenness) but a low clustering coefficient.

Supplementary Figure S2

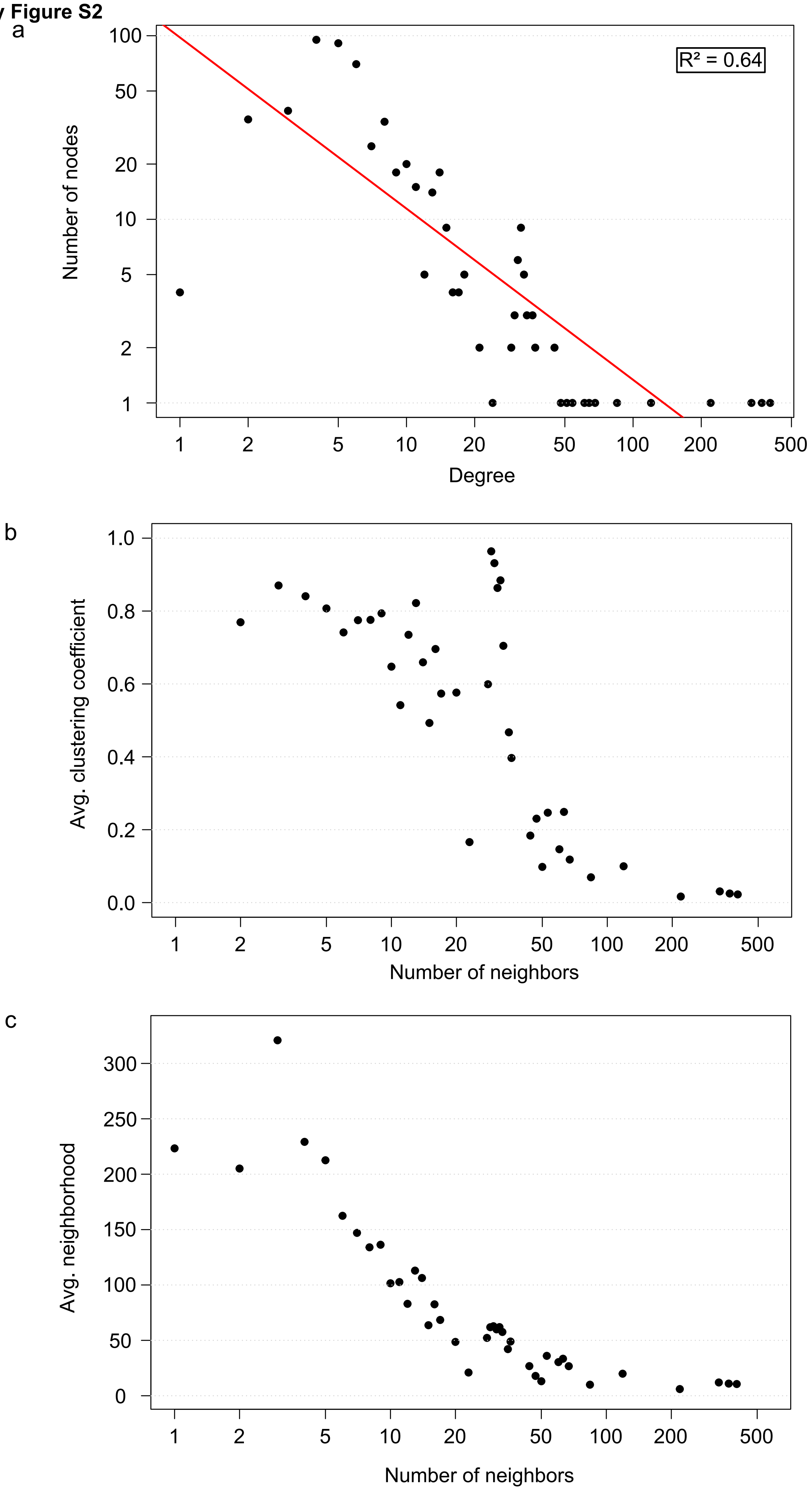

Supplementary Figure S2. Characteristics of the DIANA-microT meet/min 0.5 graph.

a. Degree distribution. There is a slight correspondence to a scale-free graph ($R^2 = 0.64$). Most nodes in the graph are low-degree nodes. Fitting was performed using a linear model on the logarithm of the data (lm function).

b. Average clustering coefficient. The clustering coefficient or transitivity ratio measures how neighbours of a node are connected. Hubs (more than ~100 neighbours) tend to connect to isolated nodes, while low degree nodes are highly clustered. These low degree and highly clustered nodes are in fact the different families that we still find at meet/min 0.5.

c. Average neighbourhood connectivity distribution. The neighbourhood connectivity is the average connectivity of all neighbours of a node. The distribution gives the average of the neighbourhood connectivities of all nodes with a certain number of neighbours (k = 1, 2, etc.). In the graph, there is a prevalence of edges between low degree nodes and hubs of the network.

Supplementary Figure S3

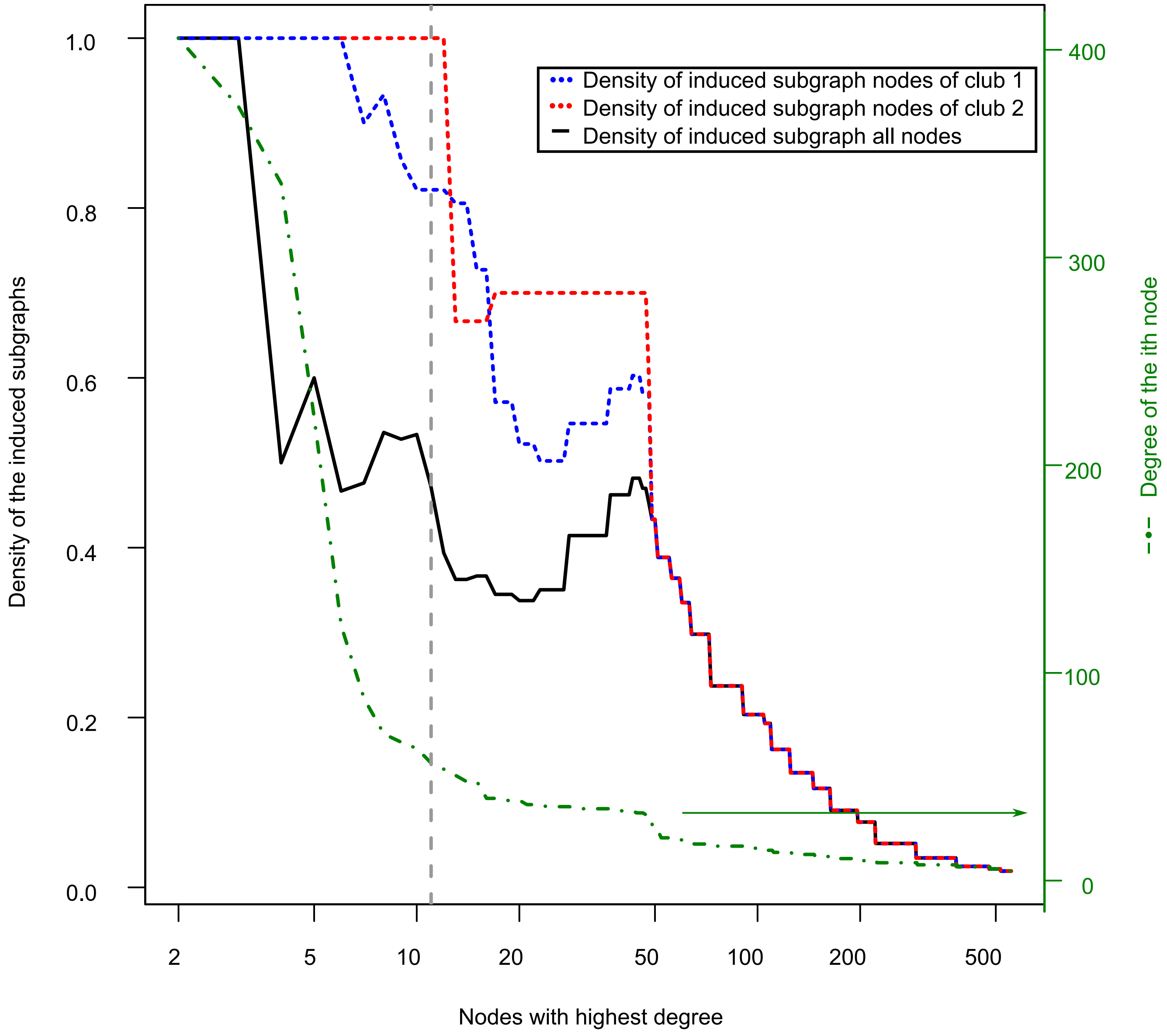

Supplementary Figure S3. Assorted club detection. Nodes are sorted from the highest to the lowest degree. At 11 microRNAs, the whole graph shows two separated assorted clubs, with an average density of 0.5. The two clubs meet to form one graph with the addition of the 48th microRNA.

Supplementary Figure S4

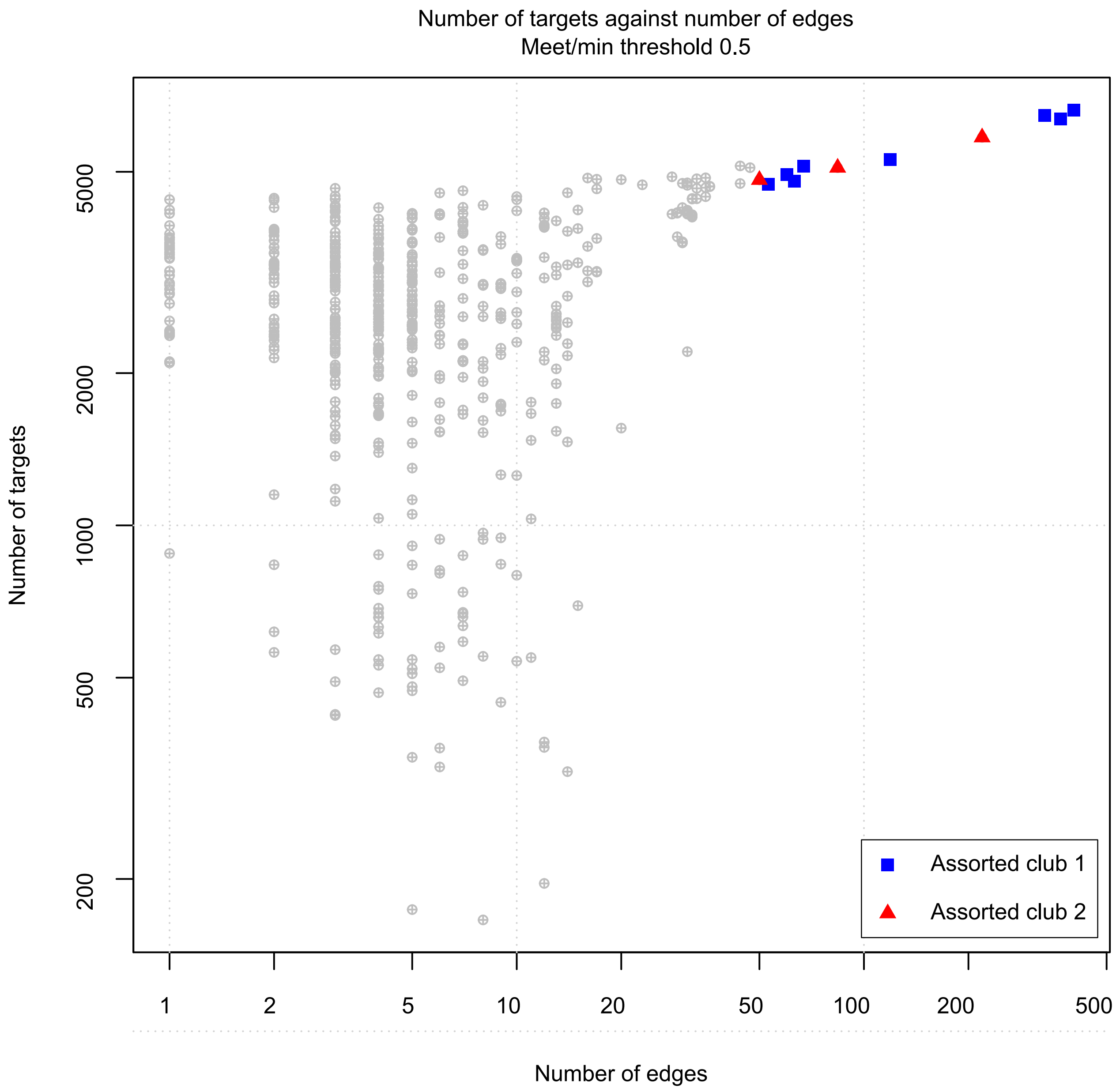

Supplementary Figure S4. Number of predicted targets versus the degree of microRNAs. There is a slight correlation between microRNAs with a high number of predicted targets and the microRNAs that have many links in the network.

Supplementary Figure S5

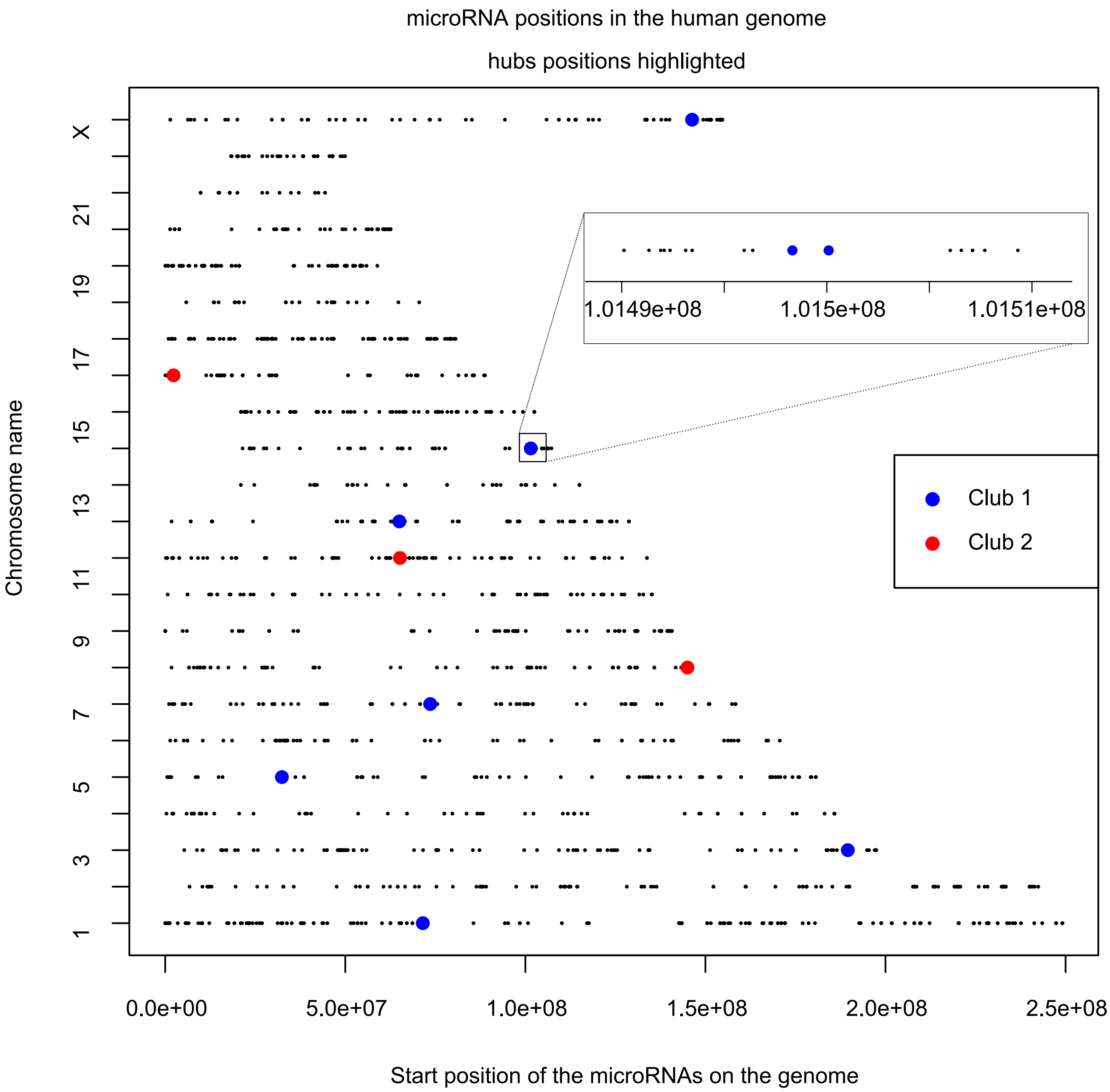

Supplementary Figure S5. microRNA positions on the genome. The position of the 11 microRNAs from the two assorted clubs are highlighted in blue and red. miR-495 and miR-543 are located on the 14th chromosome in very close proximity to each other. Apart from these two microRNAs, the hubs are equally distributed in the genome. This plot was generated using the R/Bioconductor package biomaRt.

Supplementary Figure S6

Venn diagrams of protein coverage between protein hubs, the assorted clubs and their respective sphere of influence

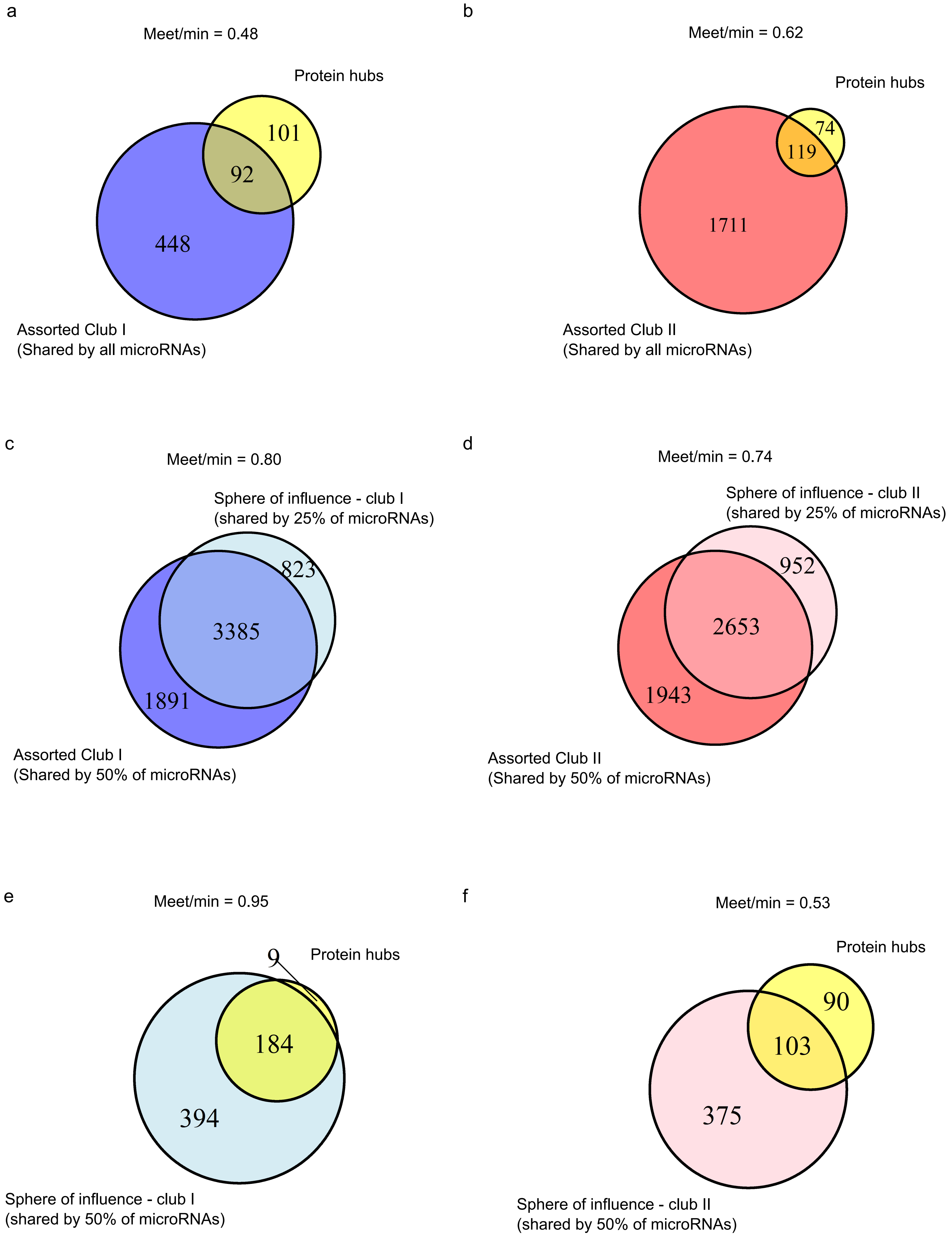

Supplementary Figure S6. Venn diagrams of target coverage between protein hubs, assorted clubs and their respective spheres of influence.

a. Coverage between targets shared by all microRNAs from assorted club 1 and the 193 protein hubs.
b. Coverage between targets shared by all microRNAs from assorted club 2 and the 193 protein hubs.
c. Coverage between targets shared by 50% of the microRNAs from assorted club 1 and targets shared by 25% of the members of its sphere of influence.
d. Coverage between targets shared by 50% of the microRNAs from assorted club 2 and targets shared by 25% of the members of its sphere of influence.
e. Coverage between targets shared by 50% of the microRNAs from the sphere of influence of assorted club 1 and the 193 protein hubs.
f. Coverage between targets shared by 50% of the microRNAs from the sphere of influence of assorted club 2 and the 193 protein hubs.

Supplementary Figure S7

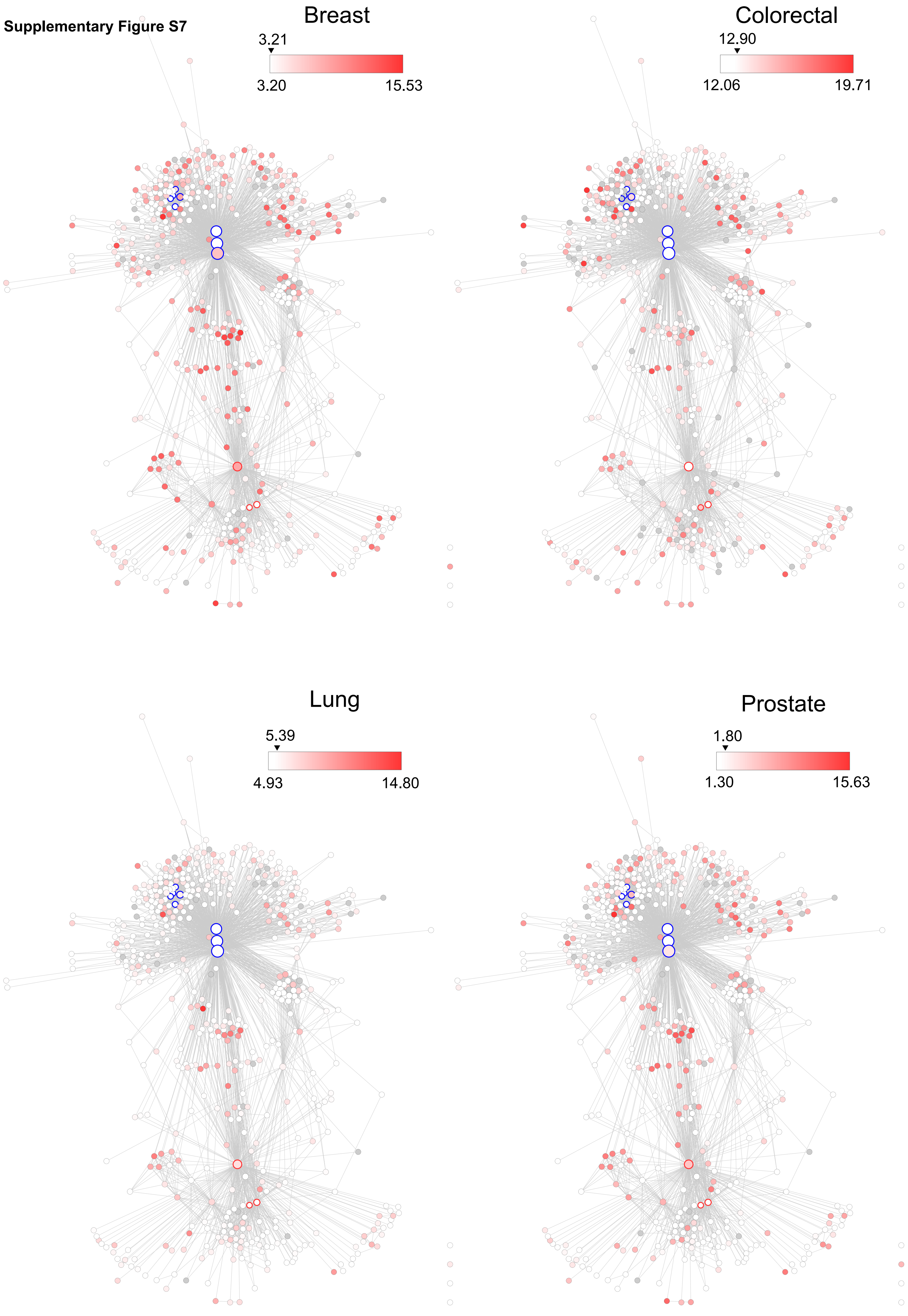

Supplementary Figure S7. DIANA-microT v3 network with microRNA expression of breast, colorectal mucosa, lung and prostate normal tissues. Are represented breast (GSE45666), colorectal mucosa (GSE38389), lung (GSE25508) and prostate (GSE34933) normal tissues. A linear gradation from white to red is used to visualize individual microRNA expressions. The gradation begins at the median observed value of all microRNAs (arrows) in the corresponding dataset and expands to the maximal observed value. The whole box expands from the minimum observed value to the maximum observed expression value in the graph. Unobserved microRNA in a dataset are coloured in grey. Highly expressed microRNAs are thus red whereas low and unexpressed microRNA are white.

Supplementary Figure S8

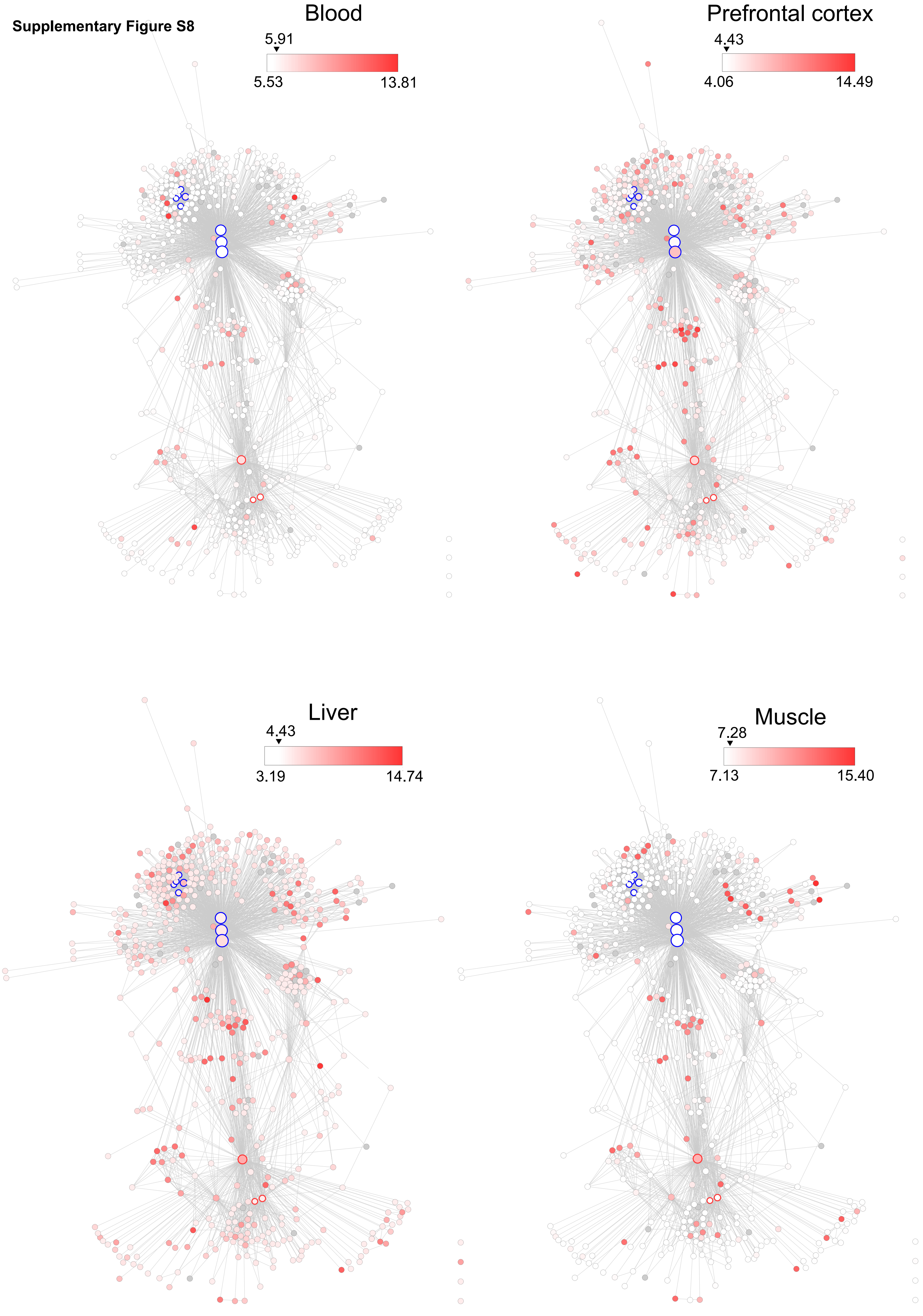

Supplementary Figure S8. DIANA-microT v3 network with microRNA expression of blood, prefrontal cortex, liver and muscle normal tissues. Are represented blood (GSE24205), prefrontal cortex (GSE19505), liver (GSE19505) and skeletal muscle (GSE23527) normal tissues. A linear gradation from white to red is used to visualize individual microRNA expressions. The gradation begins at the median observed value of all microRNAs (arrows) in the corresponding dataset and expands to the maximal observed value. The whole box expands from the minimum observed value to the maximum observed expression value in the graph. Unobserved microRNA in a dataset are coloured in grey. Highly expressed microRNAs are thus red whereas low and unexpressed microRNA are white.

Supplementary Figure S9

a

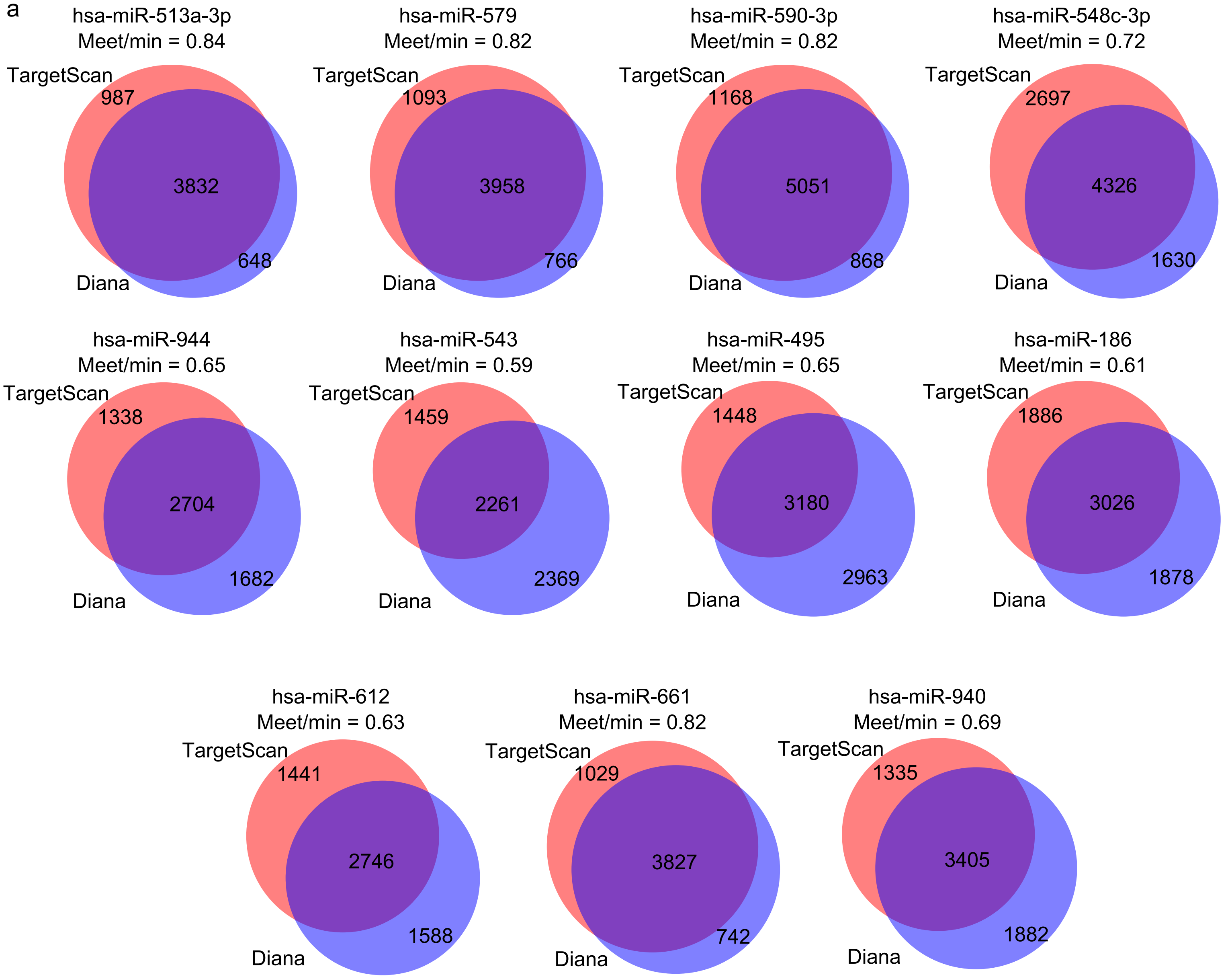

b

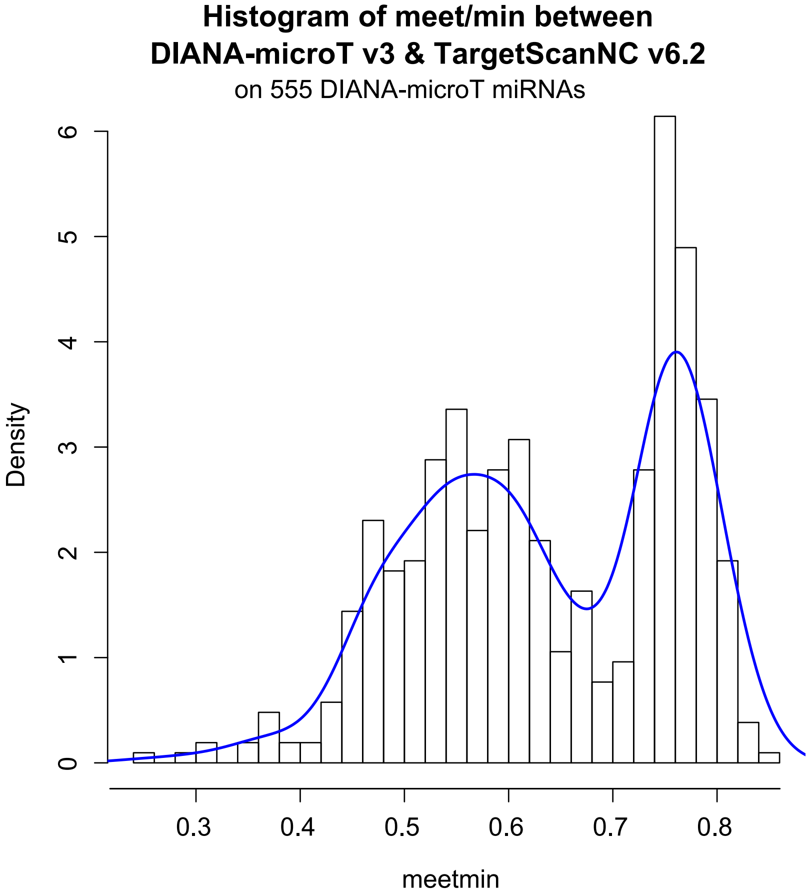

Supplementary Figure S9. Target prediction coverage between DIANA-microT v3 and TargetScan Non-Conserved v6.2.

a. Venn diagrams of coverage between the targets of the members of the assorted club 1 (blue) and 2 (pink) from DIANA-microT. The meet/min metric was used to evaluate the coverage of target prediction between the two algorithms. On average, there is coverage of 0.71. miR-513a-3p is the microRNA with the highest coverage (0.84, most of the predictions are the same), and miR-543 has the lowest coverage (0.59, more than half are identical)

b. Histogram of prediction coverage for all microRNAs from DIANA-microT v3. The histogram displays a bimodal distribution with a first population at 0.55 and a second one at approximately 0.8. The mean (and median) is equal to 0.64 (0.63), meaning that on average, 60% of predicted targets are identical between both algorithms.

Supplementary Figure S10

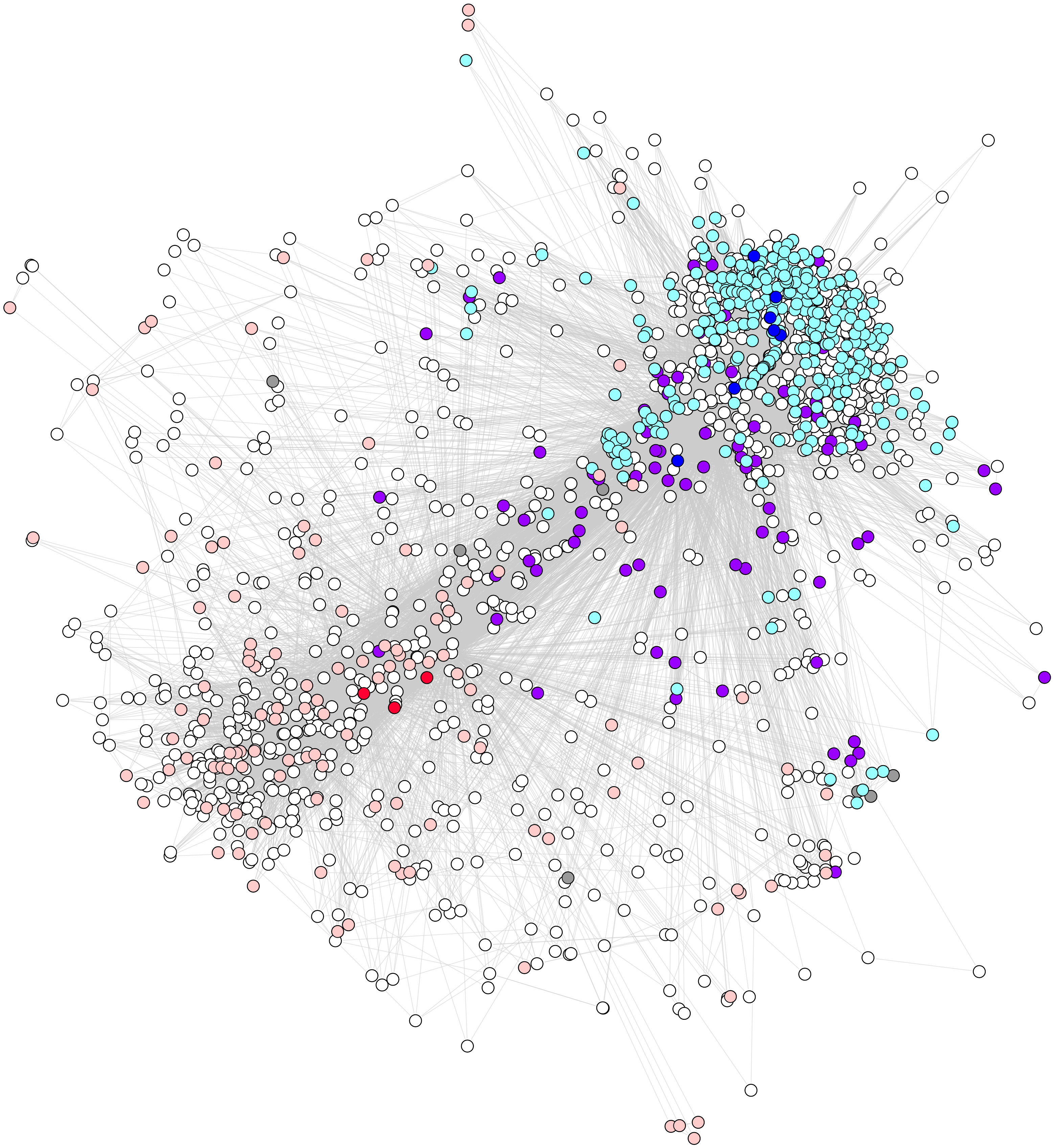

Supplementary Figure S10. TargetScan Non-Conserved network. The TargetScan Non-Conserved network was built using the same procedure as the DIANA-microT network. The colour scheme is the same as for the DIANA-microT network (cyan and pink, neighbourhood of assorted club 1 and 2, respectively; in purple, the microRNAs connected to both clubs; and in grey, microRNAs connected to no clubs). All microRNAs from TargetScan are represented here. Those absent from DIANA-microT are represented in white. The same global organization is found, meaning two distinct poles, hubs and groups of highly connected microRNA.

Supplementary Figure S11

TargetScan Non Conserved v6.2

a

Number of connected nodes and edges

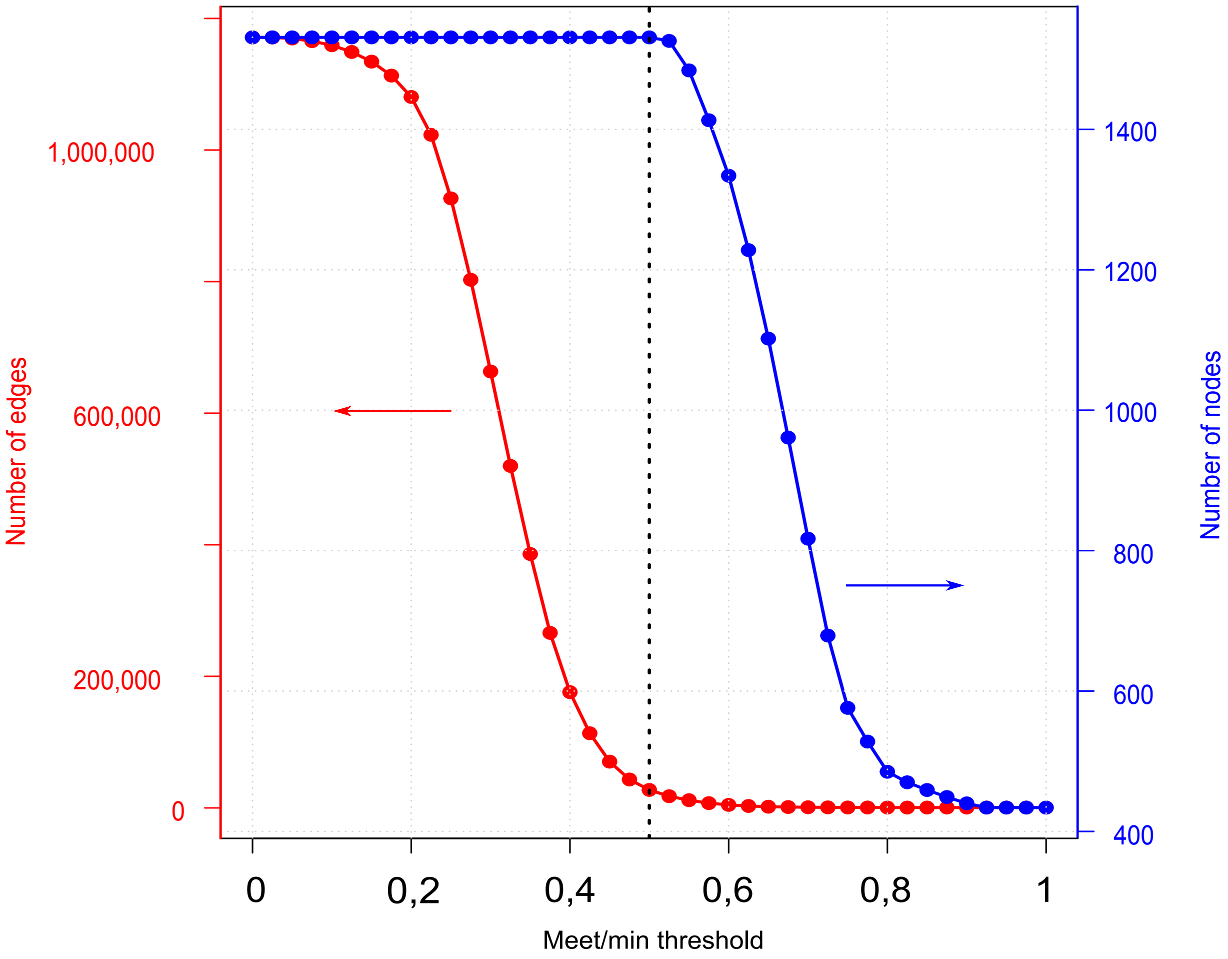

b

Centrality measurements

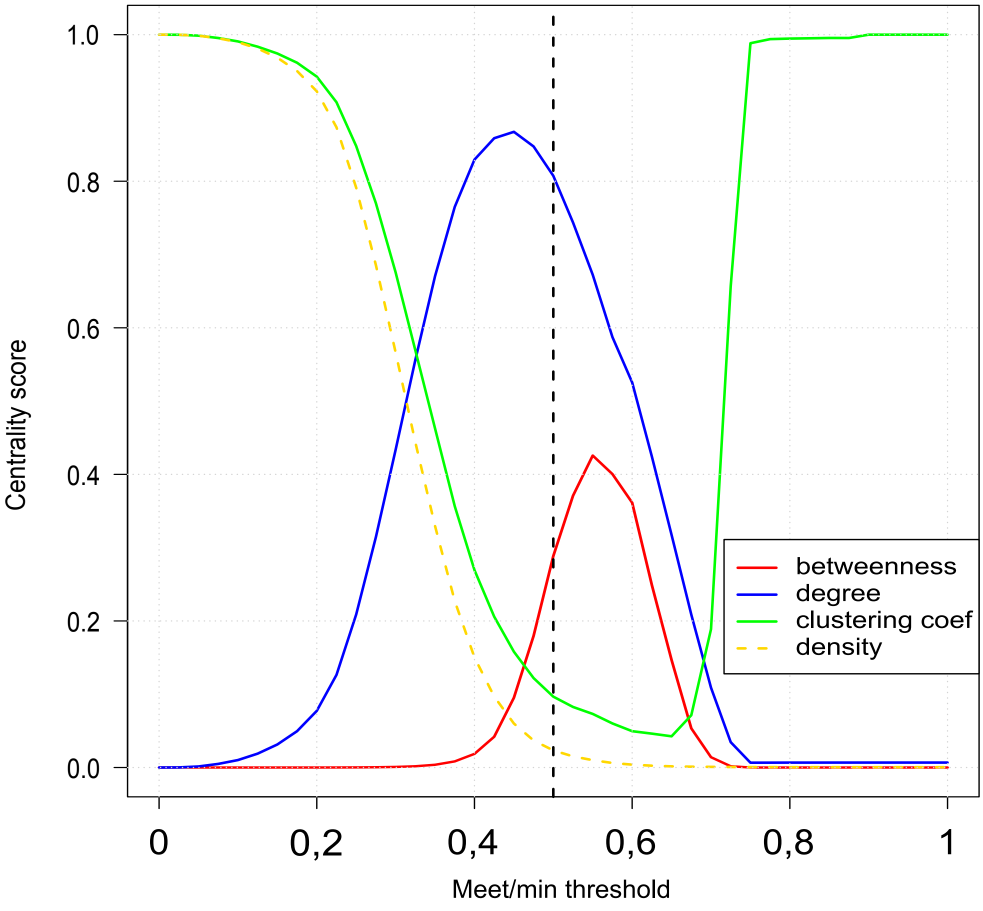

Supplementary Figure S11. Network properties of the TargetScan Non-Conserved network. These graph behaviours are highly similar to those observed using DIANA-microT (Supplementary Figure S1e)

a. Number of edges and connected nodes as a function of meet/min thresholds.
b. Density, clustering coefficient, betweenness and degree centrality measurements as a function of meet/min thresholds. The increment of the meet/min threshold is 0.05 from 0 to 1. The 0.5 meet/min threshold results in a high number of connected nodes with a low number of edges and high centrality measurements but a low clustering coefficient, as does the DIANA-microT network.

Supplementary Figure S12

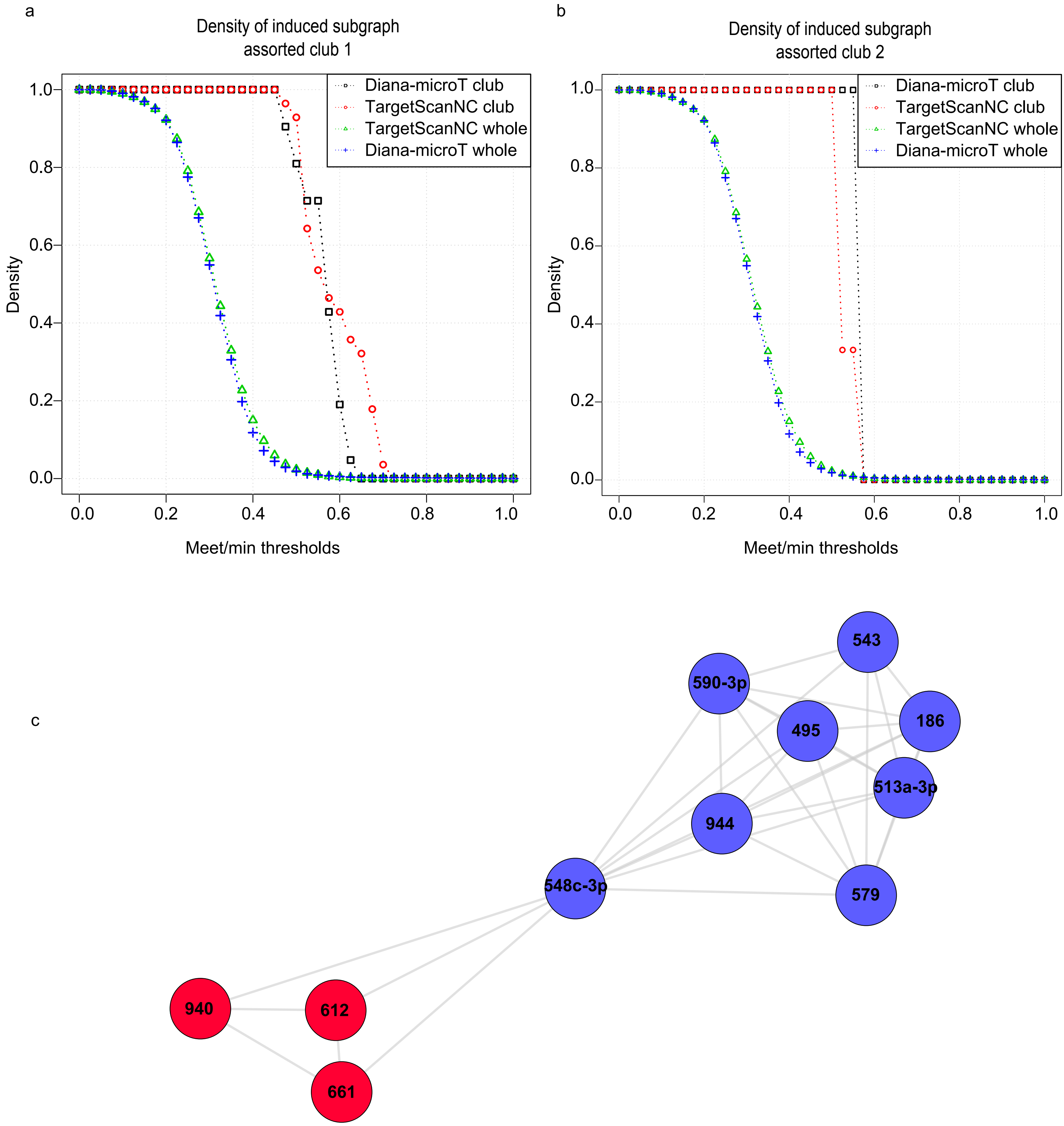

Supplementary Figure S12. Induced subgraphs and density profiles of the two assorted clubs from DIANA-microT and TargetScan Non-Conserved analysis.

a. Assorted club 1 induced subgraph density.

b. Assorted club 2 induced subgraph density.

c. Induced subgraph of the assorted clubs 1 and 2 from the DIANA-microT and TargetScan Non-Conserved networks.

Both clubs are connected in a similar way in the results from the two target prediction databases (red and black). Only slight changes can be seen. The two clubs are, however, connected through miR-548c-3p in this network. The densities of the two whole networks (in blue and green, a and b) are practically the same. The densities of the induced subgraph formed by the members of the assorted clubs 1 and 2 in this network are equal to 0.93 and 1, respectively.

Supplementary Figure S13

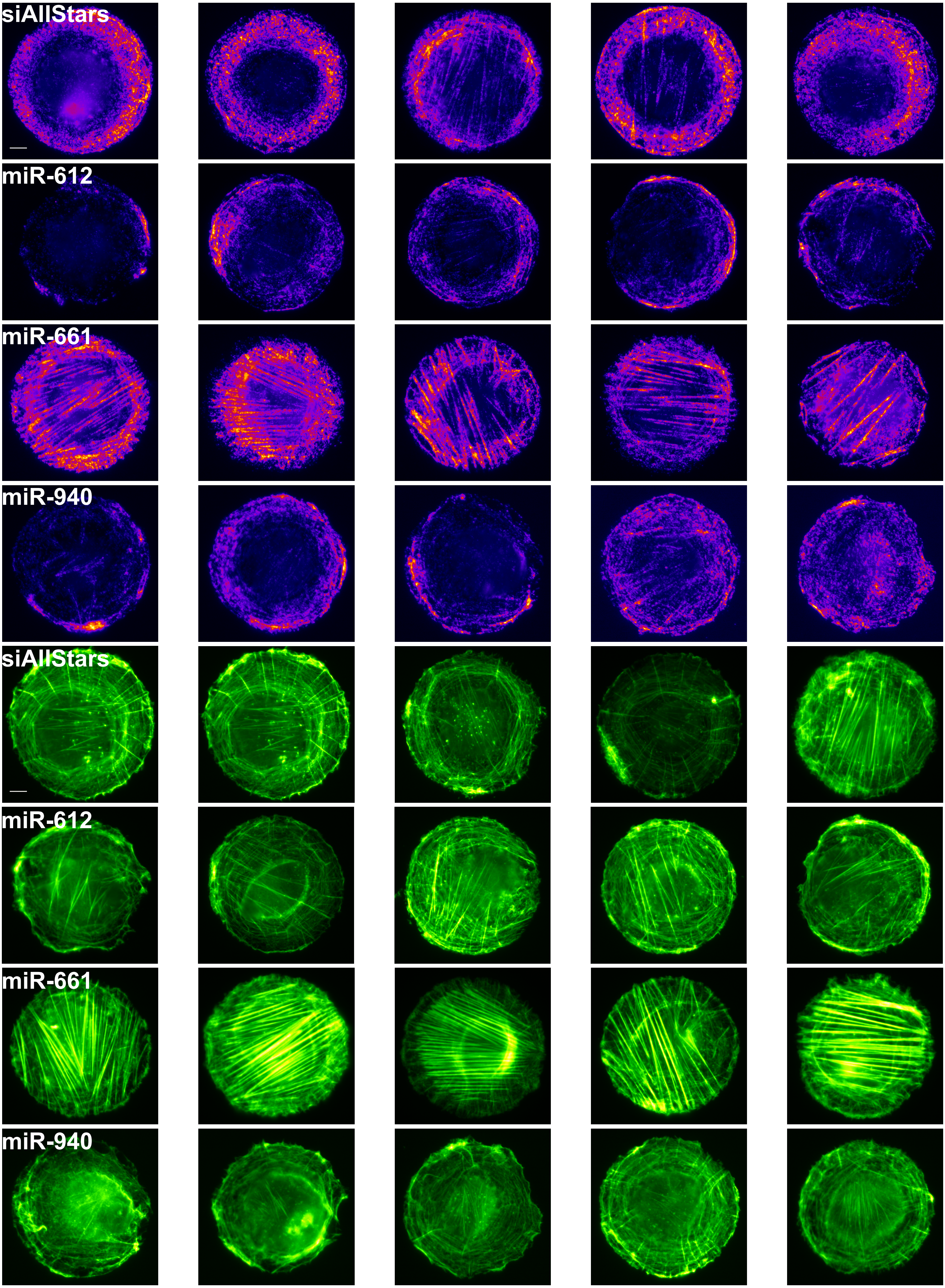

Supplementary Figure S13. Immunofluorescence in individual images of actin and myosin staining on 1000µm² circular fibronectin patterns. Images were acquired on the same day and under the same conditions, cf. Figure 3.c in the main text.

Supplementary Figure S14

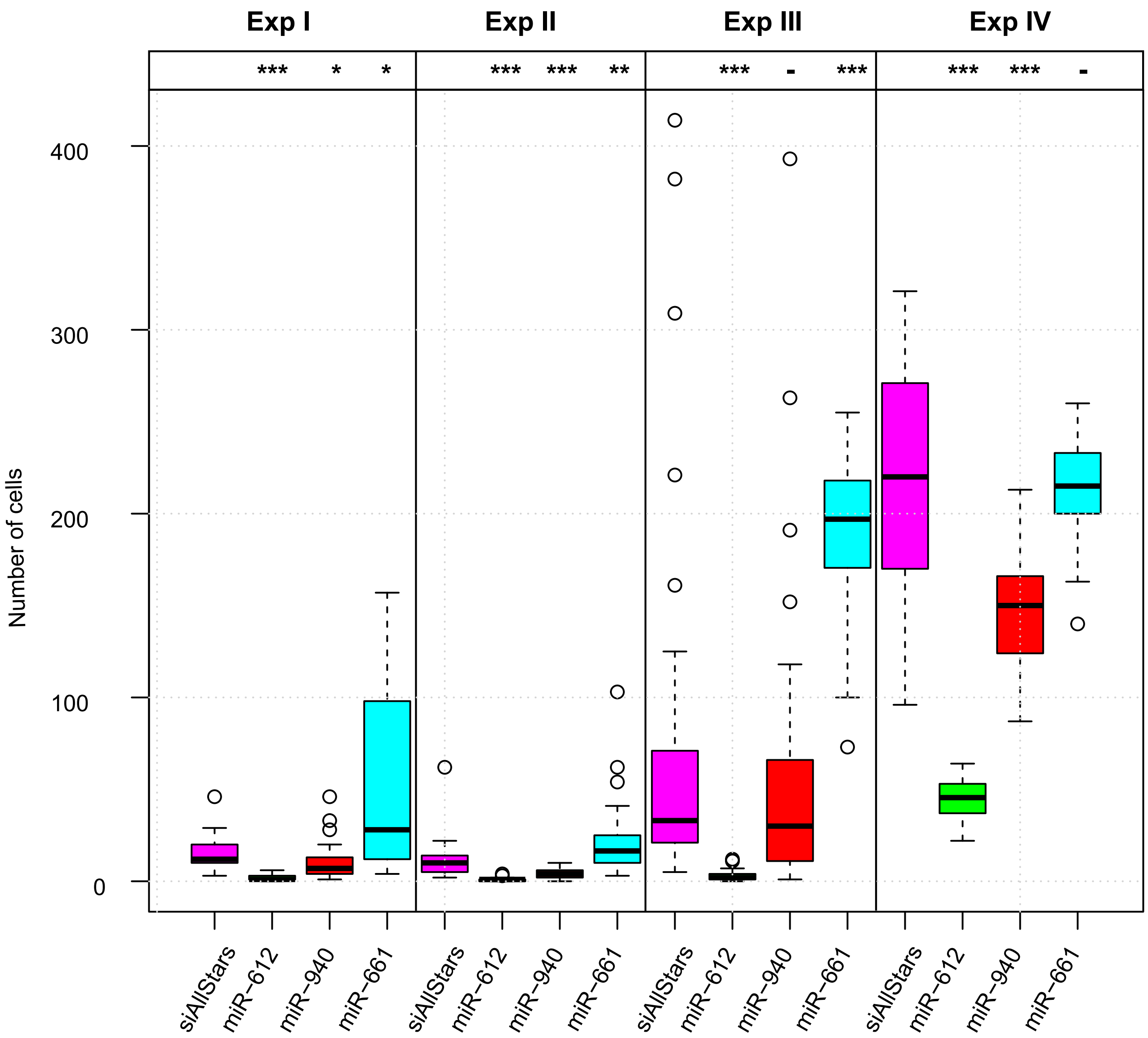

Supplementary Figure S14. Motility graph: number of cells for the transwell assays. Four independent experiments were conducted with increasing numbers of deposited cells. These increasing numbers were used to examine different phenotypes. A low number of cells allowed the identification of the microRNAs that greatly promote cell migration, while a high number of cells allowed the identification of microRNAs that inhibit cell migration. The Wilcoxon test was used on each experiment to calculate the P-values, with siRNA AllStar as a control in each case (P-value < 0.001: ***; P-value < 0.01: **; P-value < 0.05: *; P value ≥ 0.05: -). In the first experiment, 17, 30, 29, and 30 images were considered for the test. In the second experiment, 30 images were analysed for each condition. In the third experiment, 29 images for siRNA AllStar and 30 images for all three other microRNAs were used. Finally, for the final experiment, 31 images were used for miR-661, and 30 images were used for the three other conditions. This variability is essentially due to the image quality.

P - values	Exp I	Exp II	Exp III	Exp IV
miR - 661	0.015	0.0023	$1.0\ 10^{-06}$	0.98
miR - 612	$3.24\ 10^{-08}$	$5.27\ 10^{-11}$	$9.11\ 10^{-11}$	$4.43\ 10^{-11}$
miR - 940	0.023	$7.9\ 10^{-06}$	0.29	0.00011

Supplementary Figure S15

a

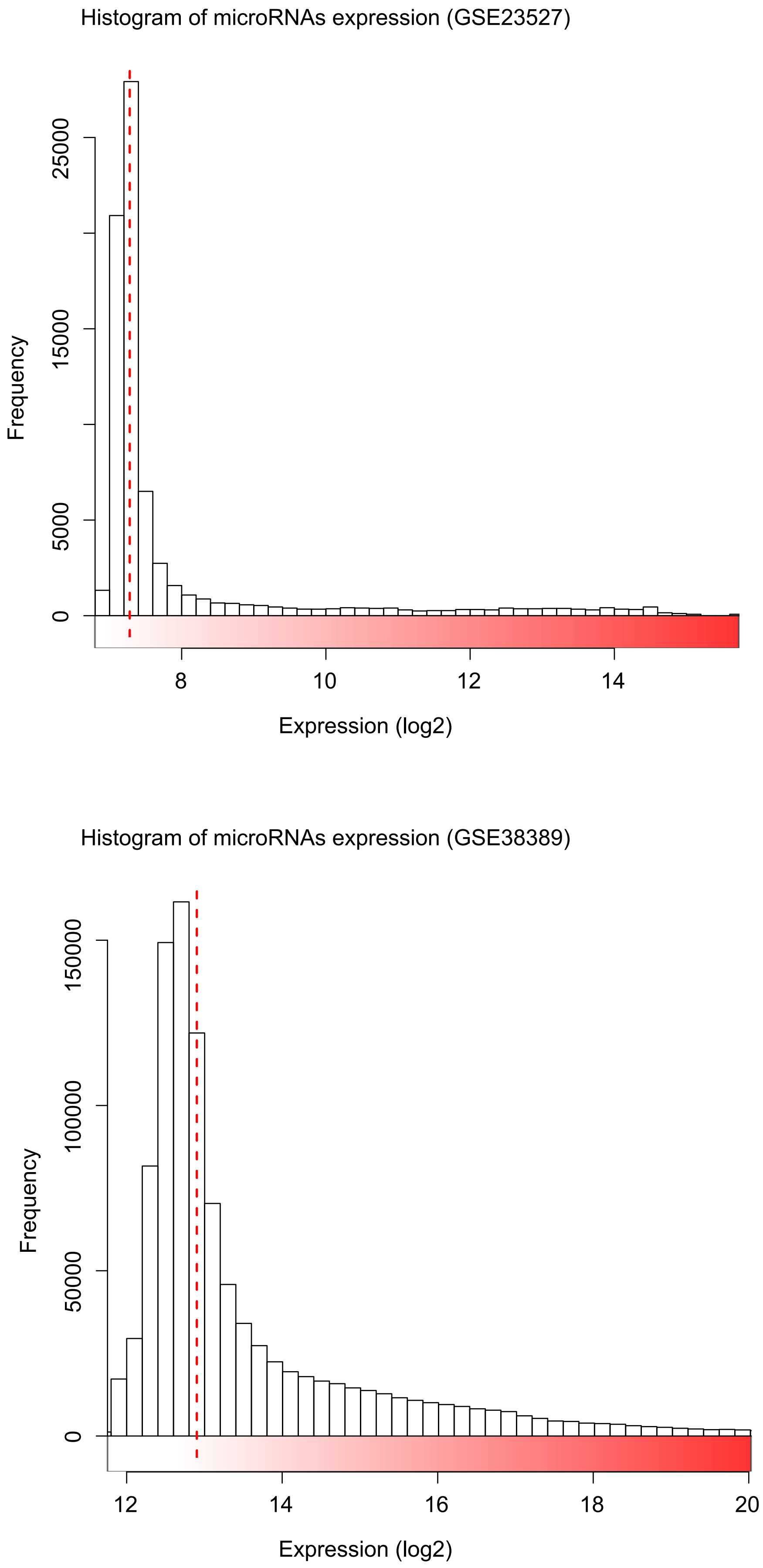

Supplementary Figure S15. Example histograms of microRNA expression.
a. Normal skeletal muscle tissue (GSE23527)
b. Normal colorectal mucosa (GSE23527).
The red dotted lines correspond to the median observed value of microRNA expression dataset (normalized but non-aggregated). The gradation is the one exposed in Supplementary Figure S13 & S14 and expand from the median to the maximum observed value. Expression below the median are all considered low or unexpressed.
